# Exercise, physical activity, and physical fitness in psoriasis and psoriatic arthritis: an umbrella review

**DOI:** 10.3389/fimmu.2026.1921728

**Published:** 2026-10-09

**Authors:** Zikun Lyu, Jingshi Ma, Chenzhe Ma, Xuechen Tian, Liye Liu, Jiayi Zhang, Yafei Gao

**Affiliations:** 1 Department of Physical Education, Kunsan National University, Gunsan-si, Republic of Korea; 2 School of Physical Education, Handan University, Handan, Hebei, China; 3 Northeast Petroleum University, Daqing, China

**Keywords:** evidence synthesis, exercise, physical activity, physical fitness, psoriasis, psoriatic arthritis, systematic review, umbrella review

## Abstract

**Background:**

Exercise, physical activity, and physical fitness are increasingly discussed in psoriasis and psoriatic arthritis (PsA), but the evidence base is uneven: intervention reviews predominantly concern PsA, whereas evidence for cutaneous psoriasis is largely observational or embedded in broader lifestyle interventions.

**Methods:**

This systematic review was conducted as an umbrella review of intervention effects and observational exposure–outcome associations. The protocol was registered in PROSPERO (CRD420261403713). PubMed, Web of Science, Embase, Cochrane Library, Scopus, and SPORTDiscus were searched from inception to 1 March 2026. Two reviewers independently screened records and extracted data, with disagreements resolved by discussion and, when necessary, a third reviewer. Reporting followed PRISMA (Preferred Reporting Items for Systematic Reviews and Meta-Analyses) 2020 and PRIOR (Preferred Reporting Items for Overviews of Reviews). AMSTAR 2 (A Measurement Tool to Assess Systematic Reviews 2), corrected covered area (CCA), GRADE (Grading of Recommendations, Assessment, Development and Evaluations), and the Ioannidis credibility classification were applied as appropriate.

**Results:**

Nine reviews were included. Eight reviews provided PsA evidence, including interventional, descriptive, or observational findings; one observational meta-analysis concerned prevalent cutaneous psoriasis. No eligible review isolated the effect of exercise on cutaneous-psoriasis severity. Overall confidence was Moderate for one review, Low for five, and Critically Low for three. No umbrella-level pooling was undertaken because the same trials recurred across reviews, particularly in the high-intensity interval-training/cardiorespiratory cluster (CCA approximately 16.7%), and populations, outcomes, comparators, and effect measures were heterogeneous. Vigorous activity (RR 0.76, 95% CI 0.67 to 0.85) and regular exercise frequency (RR 0.88, 95% CI 0.82 to 0.95) were associated with lower psoriasis prevalence, both with suggestive credibility. In PsA, possible improvements in fitness, disease activity, pain, function, and body composition were based mainly on single small trials and had very low certainty.

**Conclusion:**

The evidence differs by disease. For cutaneous psoriasis, current findings are observational and do not establish an exercise effect on disease severity or incidence. For PsA, intervention findings are sparse, duplicated across reviews, and of very low certainty. Safety reporting was incomplete and did not permit a disease-specific conclusion. Adequately powered, prospectively registered trials with disease-specific outcomes and systematic adverse-event reporting are required.

**Systematic Review Registration:**

https://www.crd.york.ac.uk/PROSPERO/, identifier CRD420261403713.

## Introduction

Psoriasis is a common, chronic, immune-mediated inflammatory disease affecting a substantial share of the adult population worldwide (1). A considerable proportion of people with psoriasis develop psoriatic arthritis (PsA), a heterogeneous musculoskeletal disease that can involve peripheral joints, the axial skeleton, entheses, and skin and nails, and that carries a meaningful burden of pain, fatigue, and functional limitation (2). Beyond the skin and joints, psoriatic disease is recognized as a systemic condition associated with substantial impairment of health-related quality of life and with a range of comorbidities (3).

Among these comorbidities, cardiovascular and cardiometabolic disease are especially prominent: psoriasis has been associated with an increased risk of major adverse cardiovascular events (4) and with a higher incidence of cardiovascular disease more broadly (5). This cardiometabolic burden provides a strong rationale for interventions, such as exercise and increased physical activity, that may influence both inflammatory and metabolic pathways. General-population guidance recommends regular physical activity for adults of all ages as a cornerstone of health promotion (6), and disease-specific recommendations endorse physical activity for people with inflammatory arthritis and osteoarthritis (7).

Exercise, physical activity, and physical fitness are distinct but related constructs. Exercise denotes planned, structured, repetitive activity undertaken to improve or maintain fitness; physical activity encompasses any bodily movement that expends energy; and physical fitness describes attributes such as cardiorespiratory capacity and muscular strength. In psoriatic disease, these constructs are of dual interest: as potential interventions that may improve symptoms, function, body composition, and fitness, and as exposures that may be associated with the risk of developing psoriasis or PsA, or with structural progression. Mechanical stress at entheses, for example, has been proposed as a factor in PsA, raising the possibility that some forms of loading could carry risk as well as benefit.

Systematic reviews have examined exercise, physical activity, and physical fitness in psoriasis and PsA, but they differ in population (PsA-specific versus mixed rheumatic and musculoskeletal disease), exposure, comparator, outcome, study design, and methodological rigor. The evidence base is also asymmetric: several reviews concern PsA or mixed inflammatory arthritis, whereas cutaneous-psoriasis evidence is largely observational or embedded in broader lifestyle interventions. A single review therefore gives only a partial view, while counting reviews without examining their primary studies can overstate the amount of independent evidence. An umbrella review (8) is useful here not to force a pooled estimate from an immature evidence base, but to separate the two diseases and evidence types, quantify repeated primary studies, appraise confidence, and identify where review-level evidence is absent. This overview followed Preferred Reporting Items for Overviews of Reviews (PRIOR) (9), Preferred Reporting Items for Systematic Reviews and Meta-Analyses (PRISMA) 2020 (10), and PRISMA-S (11).

Accordingly, this umbrella review mapped and appraised review-level evidence separately for cutaneous psoriasis and PsA. We asked: (i) whether the evidence was interventional or observational; (ii) which exercise or activity types, intensities, durations, comparators, and outcomes had been studied; (iii) how large and clinically interpretable the reported findings were; (iv) how certain and independent the evidence was; and (v) where the major evidence gaps remained. An intervention-effect branch and an observational exposure-outcome association branch were kept separate throughout.

## Methods

### Protocol, registration, and reporting

This systematic review was conducted as an umbrella review (overview of reviews) and is reported in accordance with PRISMA 2020 (10) and PRIOR (9); the literature search follows PRISMA-S (11). The protocol was registered in PROSPERO (CRD420261403713). The registered record prespecified review-level evidence on both intervention effects and observational exposure–outcome associations and a narrative umbrella synthesis, with pooled estimates reported only as presented by source reviews and no *de novo* meta-analysis of primary studies. Accordingly, the two evidence streams and the completed narrative approach without new pooling were consistent with the registered plan; only the branch labels and allocation rule were operationalized during extraction. Departures or additions were that SPORTDiscus was searched instead of the planned PsycINFO, A Measurement Tool to Assess Systematic Reviews 2 (AMSTAR 2) appraisal was performed independently by two reviewers rather than by one assessor with a second checker, and the evidence-tier classification and Ioannidis credibility framework were added after registration. Adverse events, flares, and safety were prespecified additional outcomes; because review-level harms reporting was sparse and non-standardized, they were summarized narratively rather than quantitatively. The eligibility criteria and other outcome domains were unchanged. No *de novo* pooling of primary-study effect estimates was performed.

### Eligibility criteria

Eligibility was defined using a PICOS framework. The population comprised people with psoriasis or PsA; reviews of mixed inflammatory arthritis or rheumatic and musculoskeletal disease populations were eligible only when psoriasis- or PsA-specific results were separately extractable. The intervention, exposure, or index factor comprised exercise, physical activity, and physical fitness, including active exercise components of physical therapy, high-intensity interval training (HIIT), aerobic, resistance or strengthening exercise, walking, home-based exercise, and physical-activity or fitness exposure levels. Comparators included no exercise, usual care, waiting list, lower physical activity, healthy or reference groups, pre–post comparisons, and alternative exercise formats, as reported by the included reviews. Outcomes included cutaneous-psoriasis severity (e.g., PASI), psoriasis prevalence or incidence associations, PsA disease activity (e.g., DAPSA, PASDAS, BASDAI, DAS, and patient global assessment), pain, fatigue, physical function, quality of life (e.g., DLQI and SF-36), inflammatory or imaging outcomes, body mass index and body composition, cardiorespiratory or muscular fitness, and physical-activity level. Eligible designs were systematic reviews, meta-analyses, Cochrane reviews, and umbrella or overview reviews; an observational meta-analysis was eligible for the psoriasis-prevalence association branch. Only full-text reviews published in English were eligible; no language restriction was applied at the search stage.

Narrative reviews, scoping reviews, and guideline records were not treated as formal review-level units, and mixed reviews without extractable psoriasis- or PsA-specific results were excluded from the main effect extraction. The two evidence streams—intervention effects and observational exposure–outcome associations—were kept separate at every stage.

### Information sources and search

Six bibliographic databases were searched from inception to 1 March 2026: PubMed (MEDLINE), Web of Science Core Collection, Embase, the Cochrane Library, Scopus, and SPORTDiscus, the last selected as a topic-specific database appropriate to an exercise-focused question. Concept blocks combined the population (psoriasis or PsA) with the exercise, physical-activity, and physical-fitness exposure and a systematic-review or meta-analysis design filter, using controlled vocabulary and free-text terms adapted to each interface. The full, interface-specific search strategies are provided in **Supplementary Table S1**.

### Selection and data collection

Two reviewers independently screened the retrieved records using prespecified eligibility criteria. They first screened titles and abstracts independently; full texts were then obtained for records that appeared potentially eligible or provided insufficient information, and each reviewer independently assessed full-text eligibility. Screening decisions were cross-checked. Disagreements were resolved through discussion and, if consensus could not be reached, by review and final adjudication by a third reviewer.

Two reviewers independently extracted data using a prespecified, piloted extraction form and then cross-checked their completed forms. Extracted items included review characteristics, populations, exercise or activity exposures, comparators, outcomes, effect measures and estimates, confidence intervals, heterogeneity statistics, contributing primary studies, time points, source-review certainty ratings, and primary-study risk-of-bias information as reported by the review. Differences were resolved through discussion and, when unresolved, by a third reviewer. Each estimate was labeled as source-review-reported or overview-author-derived and mapped to a standardized outcome construct and evidence tier (**Supplementary Table S2**).

### Methodological quality of the included reviews

Two reviewers independently appraised each included review with AMSTAR 2 (12), compared item-level judgements, and resolved disagreements through discussion; a third reviewer adjudicated any unresolved item. Overall confidence reflects the 16 AMSTAR 2 items and the seven critical domains (items 2, 4, 7, 9, 11, 13, and 15): one critical weakness lowers confidence to Low and more than one lowers it to Critically Low. Primary-study risk-of-bias tools used by the included reviews (for example, RoB 2 or the Newcastle–Ottawa Scale) were transcribed separately and were not conflated with the AMSTAR 2 appraisal; item-level ratings and rationale are provided in **Supplementary Table S3**.

### Overlap among primary studies

Because reviews on a shared question often include the same primary studies, overlap was quantified using the corrected covered area (CCA) (13). A study-by-review citation matrix was constructed, and the CCA was computed as (*N* − *r*)/(*r* × *c* − *r*), where *N* is the number of inclusions (filled matrix cells), *r* is the number of unique primary studies, and *c* is the number of reviews. Overlap was interpreted using established thresholds: 0%–5% slight, more than 5%–10% moderate, more than 10%–15% high, and more than 15% very high. Overlap was examined overall and within prespecified exercise or activity clusters.

### Certainty of the evidence

Certainty was characterized by evidence type and rating provenance. Grading of Recommendations, Assessment, Development and Evaluations (GRADE) methodology followed established guidance (14), RoB 2 was the relevant randomized-trial risk-of-bias framework where used by source reviews (15), and observational meta-analysis reporting was interpreted in line with MOOSE (16). Of 87 effect-level ratings, 81 were applied by the overview team and six were retained from source reviews. When an included review reported an outcome-specific GRADE rating for the same construct, comparator, and time point, that rating was extracted without alteration and labeled source-review-reported. Otherwise, two overview reviewers independently applied GRADE *de novo* to the unique outcome construct/evidence body using the review-reported primary-study risk of bias, inconsistency, indirectness, imprecision, and publication-bias information; duplicated estimates inherited the same construct-level judgement rather than being rated as independent evidence. Disagreements were resolved by discussion and, if necessary, a third reviewer. Narrative findings that could not support formal GRADE judgments received explicitly labeled descriptive, non-GRADE assessments. For pooled observational psoriasis-prevalence associations, the overview reviewers applied the Ioannidis class I–IV credibility criteria (8); unavailable bias inputs precluded higher classes. Heterogeneity was described with review-reported *I*^2^ values (17). The most conservative constituent judgment is shown when an outcome domain contains several constructs.

### Evidence tier

Before synthesis, each review was assigned using three operational criteria applied to its primary scope: (i) the review question and synthesis were specific to psoriasis or PsA rather than a mixed-disease population; (ii) exercise, physical activity, or physical fitness was the primary index construct rather than one component of a broader lifestyle or non-pharmacological review; and (iii) at least one prespecified disease-specific outcome was extractable. Reviews meeting all three criteria were core-eligible and supported the principal conclusions. Reviews with relevant subgroup data but a mixed-disease or broader-intervention scope were evidence-map-only; their estimates were retained in the evidence map but were not used to support the principal disease-specific conclusions.

### Synthesis approach

Synthesis was narrative and organized by disease, evidence type, and outcome domain. Umbrella-level pooling was not performed for four reasons: (i) the same primary trials and numerical estimates recurred across several reviews, creating non-independent data; (ii) populations ranged from PsA-specific samples to small PsA subgroups within mixed rheumatic populations; (iii) exercise modalities, comparators, intervention durations, and outcome time points differed; and (iv) outcomes and effect measures were not commensurable, including native-scale mean differences, standardized mean differences, ratios, descriptive values, and direction-only findings. Pooling review-level estimates under these conditions would create pseudo-replication and a clinically ambiguous summary. Quantitative estimates were therefore reported as provided by source reviews, with repeated primary-study estimates shown once in the main text and selected main table; all review-level rows remain in **Supplementary Table S2**. Publication bias and small-study effects were described only as reported by the source reviews. Without a valid pooled model, conventional sensitivity analyses and subgroup or meta-regression analyses were not applicable and were therefore not conducted. Certainty-rating provenance and rationale are reported in **Supplementary Table S4**. The CCA verification and near-duplicate-study what-if analysis are reported in **Supplementary Table S5**.

## Results

### Review selection

Nine reviews met the eligibility criteria. Eight reviews provided PsA evidence, including interventional, descriptive, or observational findings, and one observational meta-analysis provided cutaneous-psoriasis prevalence associations. Effect estimates were allocated to the intervention-effect or observational-association branch according to their design rather than by assigning each review exclusively to one branch. The flow of review-level records is summarized in **Figure 1**. The included reviews were those of Björk and colleagues (18), Gwinnutt and colleagues (19), Hailey and colleagues (20), Kaerts and colleagues (21), Kaerts and colleagues (22), Kessler and colleagues (23), Santos and colleagues (24), Sutherland and colleagues (25), and Zheng and colleagues (26). Four reviews were core-eligible (21–23, 26); the remaining five contributed to the evidence map only.

**Figure 1:**
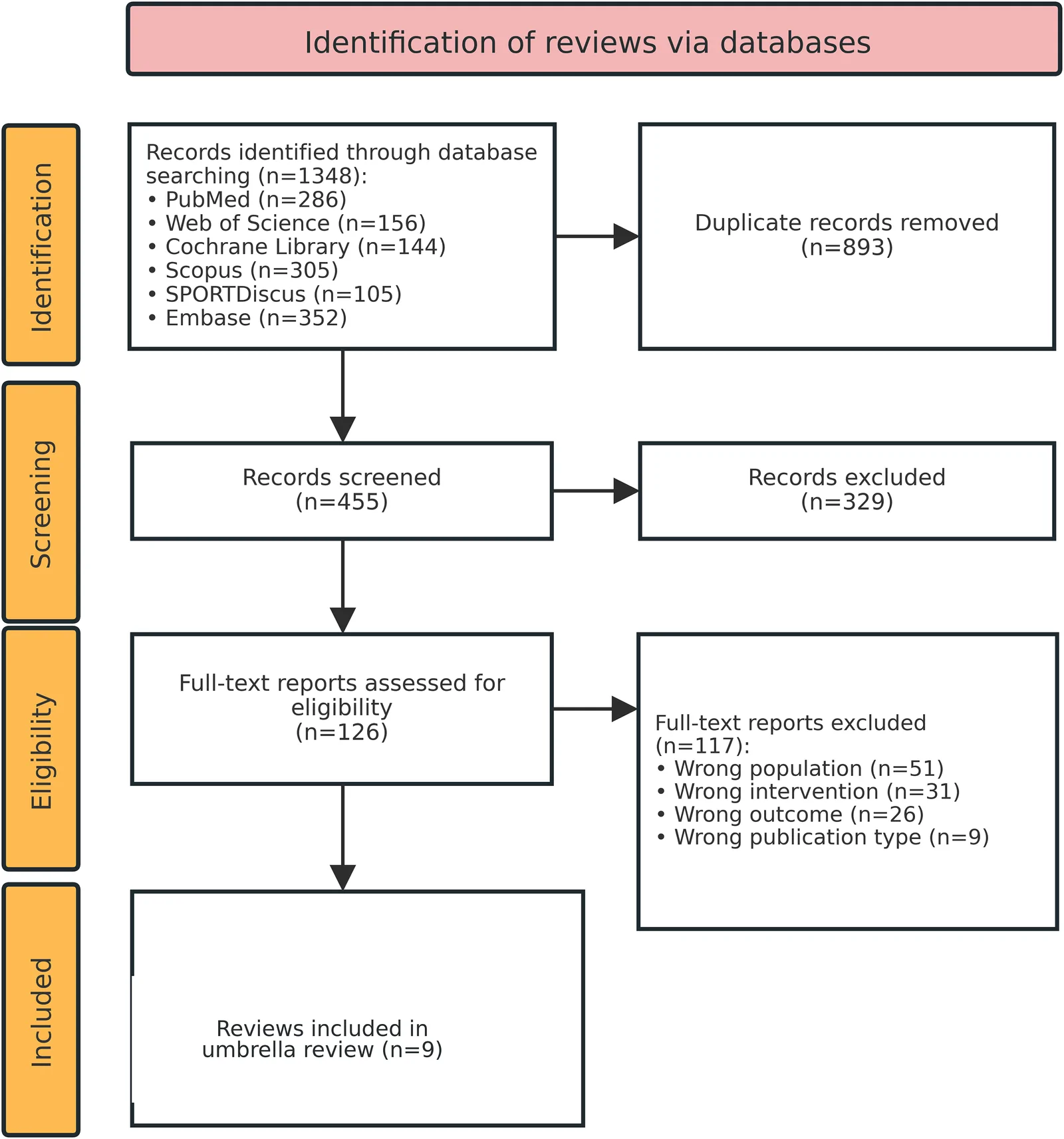
Review-selection flow diagram. Flow of review-level records through the umbrella review, following the PRISMA 2020 statement. Six bibliographic databases were searched (PubMed, Web of Science, Embase, Cochrane Library, Scopus, and SPORTDiscus) from inception to 1 March 2026. PsA, psoriatic arthritis; PRISMA, Preferred Reporting Items for Systematic Reviews and Meta-Analyses.

### Characteristics of the included reviews

The characteristics of the included reviews are presented in **Table 1**. The reviews varied from randomized-trial reviews in mixed inflammatory-arthritis populations to PsA-specific systematic reviews and one MOOSE-compliant observational meta-analysis of prevalent psoriasis. Exercise exposures included HIIT, resistance or strengthening exercise, aerobic or walking-based activity, and home exercise; observational exposures included activity intensity, frequency, and cardiorespiratory fitness. Review-level counts and randomized-trial subsets are shown in **Table 1**, with complete effect-level detail in **Supplementary Table S2**.

**Table 1:** Characteristics and disease focus of the included reviews.

Review	Review type	Population	Intervention/exposure	Studies (RCTs)	Participants	Registration	Evidence tier
Björk et al. (18)	Systematic review and meta-analysis of RCTs	Adults with inflammatory arthritis: RA, SpA, and PsA; PsA represented 4% of included studies.	Physical activity interventions including strength, aerobic, combined, and other exercise formats.	55 (55)	NR	PROSPERO CRD42020175569	Evidence-map-only
Gwinnutt et al. (19)	Systematic reviews and meta-analyses informing EULAR recommendations	People with RA, OA, SLE, axial spondyloarthritis, PsA, systemic sclerosis, or gout.	Physical exercise and body weight; only exercise/PA results relevant to PsA are extracted here.	236 exercise articles and 181 weight articles*	NR	NR	Evidence-map-only
Hailey et al. (20)	Systematic review	Adults with PsA; psoriasis/axSpA studies included only if PsA outcomes were separately reported.	Lifestyle and nonpharmacologic interventions; PA subgroup includes home exercise, resistance training, and HIIT.	8 (3)	795	PROSPERO CRD42021257404	Evidence-map-only
Kaerts et al. (21)	Systematic review	People with PsA; some studies addressed uptake, exercise interventions, therapeutic modalities, and mixed rehabilitation.	Physical therapy; cardiorespiratory training/HIIT, resistance training, therapeutic modalities, mixed rehabilitation; active exercise rows extracted here.	15 papers (10)	NR	PROSPERO 255501	Core-eligible
Kaerts et al. (22)	Systematic review	People with PsA in physical fitness and physical activity studies.	Cardiorespiratory fitness; muscular fitness/handgrip strength; objective and subjective PA; PA/fitness-related risk and disease associations.	49 papers*	10809 PsA participants in 42 unique studies	PROSPERO 255501	Core-eligible
Kessler et al. (23)	Systematic review	People with PsA; some rehabilitation studies included mixed rheumatic disease populations.	Physical activity level; HIIT; resistance/strength exercise; aerobic/strength exercise; rehabilitation programs.	13 (3 clinical trials plus additional safety/rehabilitation studies)	NR	Not registered in PROSPERO	Core-eligible
Santos et al. (24)	Systematic review and meta-analysis of RCTs	Adults with inflammatory rheumatic and musculoskeletal diseases, including PsA.	Non-pharmacological interventions; PsA-relevant row is physical activity/exercise (HIIT).	82 RCTs (82)	NR	PROSPERO CRD42021282899	Evidence-map-only
Sutherland et al. (25)	Systematic review of RCTs	Adults with inflammatory arthritis: RA, PsA, or axial spondyloarthritis.	Aerobic HIIT protocols at ≥80% HRmax with active recovery.	9 RCT articles (9)	586	PROSPERO CRD42024577039	Evidence-map-only
Zheng et al. (26)	MOOSE-compliant meta-analysis of observational studies	People with prevalent psoriasis and normal/control populations in observational studies.	Physical activity intensity, duration, exercise score, and regular exercise frequency.	13 observational studies* (0)	149,499 (17,834 psoriasis cases)	PROSPERO CRD42016045904	Core-eligible

Reviews are ordered by first-author surname (R01–R09). Core-eligible reviews had a disease-specific primary scope, treated exercise/physical activity/fitness as the primary index construct, and reported at least one extractable prespecified disease-specific outcome. Evidence-map-only reviews contributed relevant subgroup or contextual evidence but had a mixed-disease or broader-intervention primary scope. Review-level decisions are detailed in **Supplementary Table S7**. * The study/article count is summarized; full counts and randomized-trial subsets are given in **Supplementary Table S2** source notes.

CRD/PROSPERO, International Prospective Register of Systematic Reviews identifier; NR, not reported; PsA, psoriatic arthritis; RCT, randomized controlled trial; RMD, rheumatic and musculoskeletal disease.

### Distribution of evidence by disease and study design

All eight PsA-related reviews concerned PsA alone or mixed inflammatory-arthritis populations with separately extractable PsA data; none provided an exercise-only synthesis for cutaneous-psoriasis severity. Their contributions included intervention effects, descriptive fitness or activity data, and observational associations. The sole cutaneous-psoriasis review was an observational meta-analysis of prevalence associations. This disease-level separation is clinically important because PsA may remain undiagnosed among people with psoriasis (27). Among the four core-eligible reviews, three addressed PsA and one addressed psoriasis prevalence. Consequently, intervention conclusions apply to PsA, whereas the cutaneous-psoriasis conclusion is limited to observational association evidence.

### Intervention-effect branch

Selected quantitative intervention estimates are summarized by domain in **Table 2**; all extracted estimates, including direction-only findings and evidence-map-only rows, remain in **Supplementary Table S2**. Most PsA intervention results arose from two small programs: the Thomsen/Chronaiou HIIT trial family (28–31), repeatedly cited by seven reviews, and the Roger-Silva resistance-training trial (32), cited by six. Recurrent values for cardiorespiratory fitness, fatigue, and body composition therefore represent duplicate review reporting of the same participants, not replication in independent trials. Program-level trial details and review-tier decisions are provided in **Supplementary Tables S6** and **S7**, respectively.

**Table 2:** Selected quantitative estimates from core-eligible reviews, grouped by disease and outcome domain.

Review	Standardized construct	Metric	Estimate (95% CI)	Primary studies (*k*)	*I* ^2^	Analysis type
PsA disease activity—intervention evidence						
R06	Disease activity—BASDAI	—	−2/10	1	NR	Single Roger-Silva trial contrast
PsA physical function—intervention evidence						
R06	Physical function—HAQ	—	-0.27	1	NR	Single Roger-Silva trial contrast
PsA pain—intervention evidence						
R06	Pain—VAS	—	−25.1/100	1	NR	Single Roger-Silva trial contrast
PsA fatigue—intervention evidence						
R04	Fatigue—VAS	MD	−12.83 (−25.88 to 0.23)	1	NR	Single Thomsen HIIT trial estimate
PsA quality of life—intervention evidence						
R06	SF-36 bodily pain	—	72.5 vs. 53.4 at follow-up	1	NR	Single Roger-Silva trial contrast
R06	SF-36 general health	—	63.6 vs. 53.0 at follow-up	1	NR	Single Roger-Silva trial contrast
PsA cardiorespiratory fitness—intervention evidence						
R04	Cardiorespiratory fitness—VO_2_max	MD	3.72 (2.38 to 5.06)	1	NR	Single Thomsen HIIT trial estimate
R04	Cardiorespiratory fitness—VO_2_max	MD	3.08 (1.63 to 4.53)	1	NR	Single Thomsen HIIT trial estimate
PsA body composition—intervention evidence						
R04	Body composition - truncal fat percentage	MD	−1.28 (−2.51 to -0.05)	1	NR	Single Thomsen HIIT trial estimate
R04	Body composition - total body fat percentage	MD	−0.80 (−1.71 to 0.10)	1	NR	Single Thomsen HIIT trial estimate
R04	Body mass index	MD	−0.31 (−0.78 to 0.17)	1	NR	Single Thomsen HIIT trial estimate
PsA physical-activity level/behavior—descriptive evidence						
R05	Moderate-to-vigorous physical activity	—	47 (35) to 90.5 (40.8) min/day across two accelerometry studies	2	NR	Descriptive summary
R05	Physical-activity guideline attainment	—	17% to 69% meeting selected PA recommendations across studies	Multiple studies	NR	Descriptive summary
R06	Physical-activity participation	Proportion	48%; 64% of inactive avoided PA	1	NR	Descriptive summary
Cutaneous-psoriasis prevalence—observational associations						
R09	Any physical activity vs. less active	RR	0.89 (0.78 to 1.03)	13 observational studies (outcome-specific counts vary)	82%	Pooled meta-analysis (observational)
R09	Vigorous exercise vs. less active	RR	0.76 (0.67 to 0.85)	13 observational studies (outcome-specific counts vary)	0%	Pooled meta-analysis (observational)
R09	Exercise duration	SMD	−0.15 (−0.35 to 0.06)	13 observational studies (outcome-specific counts vary)	73%	Pooled meta-analysis (observational)
R09	Exercise score	SMD	0.56 (−0.89 to 2.01)	13 observational studies (outcome-specific counts vary)	98%	Pooled meta-analysis (observational)
R09	Moderate vs. strenuous activity within psoriasis	RR	0.40 (0.18 to 0.90)	13 observational studies (outcome-specific counts vary)	99%	Pooled meta-analysis (observational)
R09	Regular exercise frequency vs. less frequent	RR	0.88 (0.82 to 0.95)	13 observational studies (outcome-specific counts vary)	0%	Pooled meta-analysis (observational)
PsA incidence/structural findings—observational associations						
R05	Lower cardiorespiratory fitness and incident PsA	HR	1.44 (1.28 to 1.63)	1	NR	Single cohort study (as reported by review)
R05	Lower cardiorespiratory fitness and incident PsA	HR	1.19 (1.13 to 1.26)	1	NR	Single cohort study (as reported by review)
R05	Lower physical activity and incident PsA	HR	1.45 (0.98 to 2.13)	1	NR	Single cohort study (as reported by review)
R05	Occupational heavy lifting and PsA onset	OR	2.80 (1.51 to 5.05)	1	NR	Single primary study (as reported)
R05	Regular exercise and Achilles tendon findings	OR	1.92 (1.16 to 3.17)	1	NR	Single primary study (as reported)
R05	Physical activity and peripheral radiographic damage	Beta	0.16	1	NR	Single primary study (as reported)

This table presents selected quantitative estimates and removes duplicate review reports of the same primary-study value. Direction-only or NR rows, duplicate numerical rows, descriptive secondary contrasts, and all evidence-map-only estimates remain in **Supplementary Table S2**. The Thomsen/Chronaiou HIIT trial family underlies the cardiorespiratory-fitness, fatigue, and body-composition rows; the Roger-Silva trial underlies the resistance-training clinical rows. Estimates are reproduced as reported by source reviews; no umbrella-level pooling was performed.

CI, confidence interval; HR, hazard ratio; *I*^2^, proportion of variability across studies attributable to heterogeneity; *k*, number of contributing primary studies; MD, mean difference; NR, not reported; OR, odds ratio; RR, relative risk/ratio as reported by the source review; SMD, standardized mean difference; VO_2_max/VO_2_peak, maximal/peak oxygen uptake.

Exercise programs studied: The linked HIIT reports used the same 11-week parent protocol (NCT02995460) delivered three times weekly: a 10-minute warm-up followed by four 4-minute intervals at 85–95% of maximal heart rate, separated by 3-minute active-recovery periods at approximately 70%; two weekly sessions were supervised on a stationary bicycle and one was self-directed (28–31). The Roger-Silva (32) trial (NCT02598739) used progressive resistance exercise twice weekly for 12 weeks against conventional treatment/wait-list control (32). The distinct Silva (33) trial (NCT04304326) compared elastic-band functional exercise with machine-based resistance exercise twice weekly for 12 weeks, using three sets of 12 repetitions (33). Home-exercise evidence was less completely described. **Supplementary Table S6** provides program-level and trial-identity details. Published minimal-important-difference thresholds for BASDAI, PsA pain/fatigue, and HAQ were used for clinical interpretation (34–36).

Cardiorespiratory fitness: The HIIT trial reported increases in maximal oxygen uptake versus a no-change control (MD 3.72, 95% CI 2.38 to 5.06; and MD 3.08, 95% CI 1.63 to 4.53 for a second contrast), repeated across core-eligible reviews (21, 23). Descriptive peak oxygen-uptake values were also reported (22). The most conservative certainty was Very Low.

Body composition: The same trial reported reductions in truncal fat percentage (MD −1.28, 95% CI −2.51 to −0.05) and total body fat percentage (MD −0.80, 95% CI −1.71 to 0.10, CI crossing the null), and a small change in body mass index (MD −0.31, 95% CI −0.78 to 0.17, CI crossing the null) (21, 23). Certainty was Very Low.

Fatigue: The HIIT trial reported a fatigue visual-analog-scale change of −12.83 mm (95% CI −25.88 to 0.23) (21, 23). Although the point estimate exceeds a reported PsA minimal important difference of approximately 8.2 mm (35), the confidence interval crosses the null; a clinically important benefit is therefore not established. Certainty was Very Low.

Disease activity, pain, and physical function: These outcomes were reported mainly as single-trial contrasts or narratively. The Roger-Silva resistance-training trial reported changes of approximately −2 points on the 0–10 BASDAI, −25.1 mm on a 0–100 pain visual analogscale, and −0.27 on the HAQ relative to usual care (23, 32). The BASDAI change exceeds the approximately 1-point minimal important change established in ankylosing spondylitis, but BASDAI is not PsA-specific (34). The pain change exceeds a PsA anchor-based minimal important difference of approximately 9.4 mm, whereas the HAQ change exceeds one anchor-based estimate (−0.131) but not a more conservative trial-derived estimate (about −0.35) (35, 36). Thus, the pain and disease-activity changes may be clinically noticeable, while the functional change is threshold-dependent; all remain provisional because they derive from one small trial with Very Low certainty.

Quality of life, muscular fitness, and physical-activity behavior: Quality-of-life outcomes were reported as single-trial follow-up contrasts or narratively (23). Muscular fitness was summarized descriptively, including a range of handgrip-strength values across studies (22, 23), and physical-activity behavior was reported descriptively, with the proportion of patients meeting physical-activity recommendations varying widely across studies (22, 23). Certainty was Low to Very Low. Evidence-map-only reviews contributed additional native-scale estimates (for example, SF-36 component scores and an activity-performance standardized mean difference) that are reported in **Supplementary Table S2** but do not support the main conclusions (18–20, 24, 25).

Safety and adverse events: Safety reporting was sparse and inconsistent. In the PsA-specific lifestyle review, six of eight included studies did not report adverse events; the Roger-Silva resistance-training trial reported none. In the HIIT trial, one participant withdrew after experiencing a stroke and reported that the program was too difficult; the source review did not establish causal attribution, and no other adverse events were reported (20). Kessler et al. reported no exercise-related adverse events or inflammatory flares in the two more recent PsA trials, but an older mixed PsA/recent-onset rheumatoid-arthritis trial recorded temporary inflammatory flares in both the exercise and control groups (six versus five participants) and acute low-back pain in one participant per group (23). Safety findings from mixed inflammatory-rheumatic-disease reviews were not separately attributable to PsA (24, 25). The available review-level data therefore do not permit comparative risk estimates or a disease-specific safety conclusion. Review-level safety extraction is summarized in **Supplementary Table S8**.

### Observational exposure–outcome association branch

For cutaneous psoriasis, the only eligible disease-specific synthesis was an observational meta-analysis of prevalent disease (26). Vigorous exercise (RR 0.76, 95% CI 0.67 to 0.85; *I*^2^ = 0%) and regular exercise frequency (RR 0.88, 95% CI 0.82 to 0.95; *I*^2^ = 0%) were associated with lower psoriasis prevalence and reached suggestive (class III) credibility. The any-activity estimate was not statistically significant and was heterogeneous (RR 0.89, 95% CI 0.78 to 1.03; *I*^2^ = 82%). A within-psoriasis intensity comparison was nominally significant (RR 0.40, 95% CI 0.18 to 0.90) but extremely heterogeneous (*I*^2^ = 99%) and had only weak (class IV) credibility. Exercise-duration and exercise-score analyses were not significant. Because the underlying evidence was observational and concerned prevalence rather than incident disease, these findings do not establish that physical activity prevents psoriasis or improves skin severity.

For PsA, one systematic review summarized individual cohort associations (22). Lower cardiorespiratory fitness was associated with higher incident PsA in two estimates (HR 1.44, 95% CI 1.28 to 1.63; and HR 1.19, 95% CI 1.13 to 1.26), whereas lower-activity estimates were not statistically significant (for example, HR 1.45, 95% CI 0.98 to 2.13). Occupational heavy lifting was associated with PsA onset (OR 2.80, 95% CI 1.51 to 5.05), and regular exercise was associated with Achilles tendon findings (OR 1.92, 95% CI 1.16 to 3.17) and a small increase in peripheral radiographic damage. These Low-to-Very-Low-certainty, single-cohort findings are hypothesis-generating and do not justify restricting physical activity.

### Direct clinical summary

At review level, no exercise intervention effect has been demonstrated for cutaneous psoriasis; the available psoriasis evidence concerns observational prevalence associations. In PsA, the principal programs were 11-week HIIT and 12-week resistance or functional training, with outcomes spanning cardiorespiratory fitness, body composition, disease activity, pain, fatigue, function, quality of life, and strength. Potential benefits arose mainly from two small parent trials repeatedly represented across reviews, and safety reporting was insufficient for a conclusion. The principal gaps are exercise-only trials for cutaneous psoriasis and adequately powered PsA trials with standardized disease-specific outcomes, reproducible dosing, and complete harms reporting.

### Methodological quality

AMSTAR 2 appraisals are summarized in **Table 3**, with item-level ratings in **Figure 2** and rationale in **Supplementary Table S3**. Overall confidence was Moderate for one review (18), Low for five (20–22, 24, 25), and Critically Low for three (19, 23, 26). Eight of nine reviews did not provide a citation-level list of excluded studies with reasons; Björk et al. (18) was the exception and had no failed critical AMSTAR 2 domain. Additional critical weaknesses among the Critically Low reviews included absent prospective registration, a limited search, no formal risk-of-bias tool, or inadequate integration of risk of bias and publication bias. These limitations reduce confidence in the review-level conclusions. The domain-level certainty summary is presented in **Table 4** and interpreted below.

**Table 3:** Methodological quality of the included reviews (AMSTAR 2) and critical-domain assessment.

Review	Overall confidence (AMSTAR 2)	Critical domains not satisfied	Primary-study risk-of-bias tool(s)	Evidence tier
Björk et al. (18)	Moderate	None of the seven critical domains failed	Cochrane RoB 2.0	Evidence-map-only
Gwinnutt et al. (19)	Critically Low	I2 (no protocol/registration); I7 (no excluded-study list)	AMSTAR 2 for reviews; abbreviated Cochrane RoB tool for RCTs; QUIPS for observational studies	Evidence-map-only
Hailey et al. (20)	Low	I7 (no excluded-study list)	RoB 2 for RCTs; ROBINS-I planned but CASP cohort checklist used for NRS	Evidence-map-only
Kaerts et al. (21)	Low	I7 (no excluded-study list)	RoB 2; Newcastle–Ottawa Scale; modified RoB 2 domains for single-group interventions	Core-eligible
Kaerts et al. (22)	Low	I7 (no excluded-study list)	Newcastle–Ottawa Scale; Mendelian-randomization appraisal as reported	Core-eligible
Kessler et al. (23)	Critically Low	I2 (no protocol/registration); I4 (limited search); I7 (no excluded-study list); I9 (no formal RoB tool)	NR	Core-eligible
Santos et al. (24)	Low	I7 (no excluded-study list)	Cochrane risk of bias tool	Evidence-map-only
Sutherland et al. (25)	Low	I7 (no excluded-study list)	Cochrane RoB 2.0; PEDro scale	Evidence-map-only
Zheng et al. (26)	Critically Low	I4 (search limitations); I7 (no excluded-study list); I13 (limited RoB integration); I15 (publication bias not formally assessed)	Newcastle–Ottawa Scale (NOS)	Core-eligible

Overall confidence in the results of each review was rated with AMSTAR 2 (A Measurement Tool to Assess Systematic Reviews, version 2). The rating reflects weaknesses in the seven critical domains (items 2, 4, 7, 9, 11, 13, and 15): one critical weakness lowers confidence to Low and more than one to Critically Low. Primary-study risk-of-bias tools are those each review applied to its own included studies, transcribed from the source reports; they are distinct from the AMSTAR 2 appraisal of the reviews themselves. Item-level AMSTAR 2 ratings are provided in **Supplementary Table S3**.

AMSTAR 2, A Measurement Tool to Assess Systematic Reviews 2; NOS, Newcastle–Ottawa Scale; NR, not reported; QUIPS, Quality In Prognosis Studies; RoB 2, Cochrane risk-of-bias tool for randomized trials, version 2; ROBINS-I, Risk Of Bias In Non-randomized Studies of Interventions.

**Figure 2:**
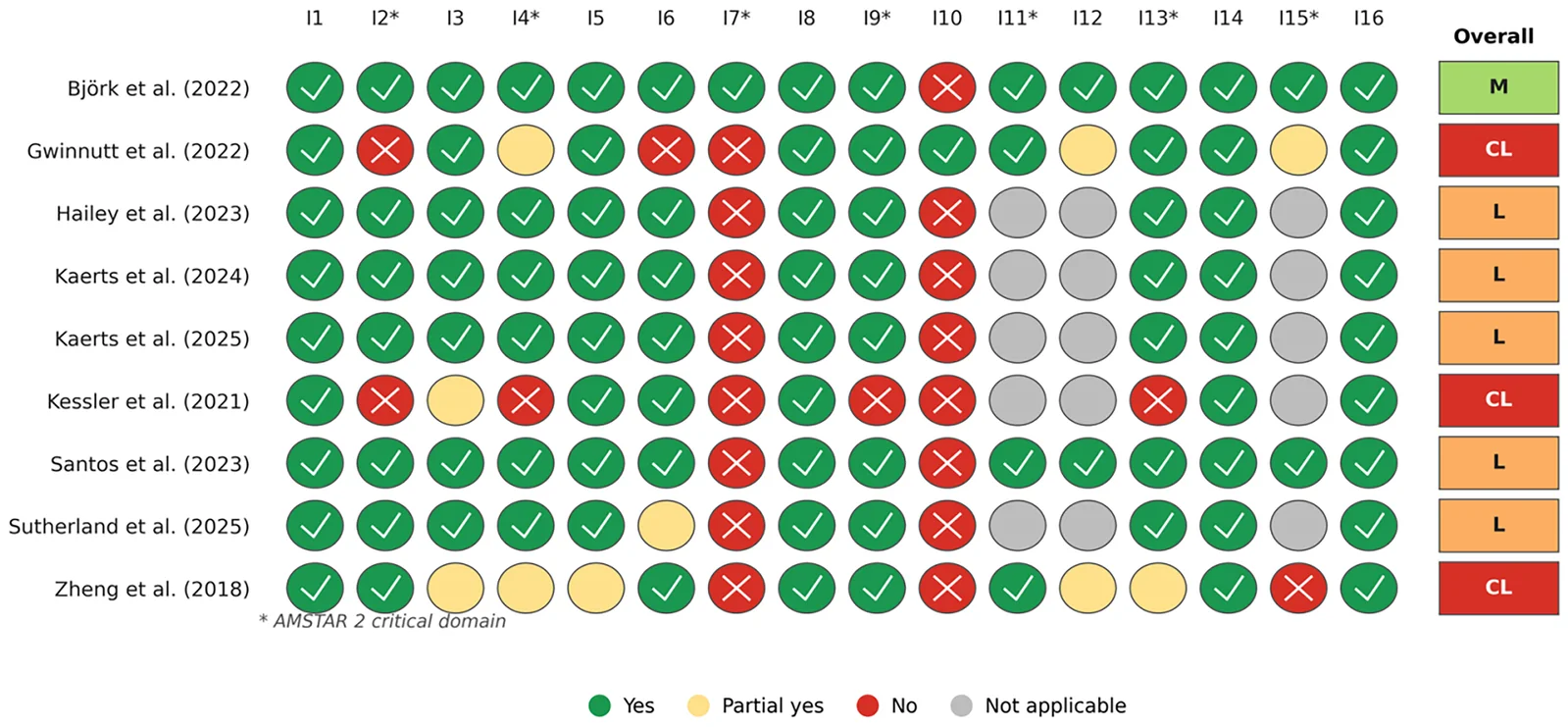
Methodological quality of the included reviews (AMSTAR 2). Item-level AMSTAR 2 ratings for each included review (rows identified by author and year) across the 16 AMSTAR 2 items (columns I1–I16), with the overall confidence rating shown at the right. Green = yes; amber = partial yes; red = no; gray = not applicable. Starred columns are the seven critical domains (items 2, 4, 7, 9, 11, 13, and 15); weakness in these domains drives the overall confidence rating. Items: I1, population–intervention–comparator–outcome; I2, protocol registered before commencement; I3, explanation of study-design selection; I4, comprehensive literature search; I5, duplicate study selection; I6, duplicate data extraction; I7, list of excluded studies with justification; I8, description of included studies; I9, risk-of-bias assessment; I10, sources of funding of included studies; I11, appropriate statistical/meta-analytic methods; I12, effect of risk of bias on synthesis; I13, risk of bias considered in interpretation; I14, explanation of heterogeneity; I15, investigation of small-study/publication bias; I16, conflicts of interest. AMSTAR 2, A Measurement Tool to Assess Systematic Reviews, version 2.

**Table 4:** Certainty of the evidence by outcome domain.

Outcome domain	Branch	Certainty framework	Certainty (range)	Most conservative	Constructs/reviews	Cross-review divergence
Quality of life	Intervention	GRADE; descriptive (non-GRADE)	Very Low	Very Low	8/4	No
Physical function	Intervention	GRADE; descriptive (non-GRADE)	Low–Very Low	Very Low	3/5	Yes
Disease activity	Intervention	GRADE	Low–Very Low	Very Low	6/5	Yes
Pain	Intervention	GRADE	Low–Very Low	Very Low	1/4	Yes
Fatigue	Intervention	GRADE	Low–Very Low	Very Low	1/5	Yes
Cardiorespiratory fitness	Intervention	GRADE; descriptive (non-GRADE)	Moderate–Very Low	Very Low	2/5	Yes
Body composition	Intervention	GRADE	Low–Very Low	Very Low	3/4	Yes
PsA incidence/structural associations	Association	Descriptive (non-GRADE)	Low–Very Low	Very Low	3/1	Yes
Muscular fitness	Intervention	GRADE; descriptive (non-GRADE)	Low–Very Low	Very Low	2/2	Yes
Physical activity level/behavior	Intervention	Descriptive (non-GRADE)	Low–Very Low	Very Low	4/2	Yes
Inflammation/imaging	Intervention	GRADE	Very Low	Very Low	2/1	No
Psoriasis prevalence associations	Association	Ioannidis class (observational)	Class III; Class IV; No class (*p* ≥ 0.05)	No class (*p* ≥ 0.05)	7/1	No

Certainty is summarized at the outcome-domain level. Of 87 effect-level ratings, 81 were overview-applied and six were source-review-reported. Source-review GRADE ratings were retained only when they matched the same construct, comparator, and time point; otherwise, two overview reviewers applied GRADE *de novo* to the unique construct/evidence body, with duplicated review reports inheriting the same judgment. Narrative findings received labeled descriptive, non-GRADE judgments. The overview reviewers applied Ioannidis class I–IV criteria to observational psoriasis–prevalence associations. Rating provenance and rationale are reported in **Supplementary Table S4**. Where a domain contained several constructs, the range and most conservative judgment are shown.

GRADE, Grading of Recommendations, Assessment, Development and Evaluation; PsA, psoriatic arthritis.

### Overlap among primary studies

Primary-study overlap by cluster is reported in **Table 5**, **Figure 3**, and **Supplementary Table S5**. Across all nine reviews, overlap was slight (CCA ≈ 2.7%; 311 unique studies across 378 inclusions), but this overall value concealed concentrated duplication. The HIIT/cardiorespiratory cluster had very high overlap (CCA ≈ 16.7%) because four reports—Thomsen (28), Thomsen (29), Chronaiou (30), and Thomsen (31)—were linked to the same Norwegian PsA parent trial (NCT02995460) (28–31) and appeared across seven reviews; differing sample sizes reflected outcome-specific analytic subsets. The resistance/strength cluster had moderate overlap (CCA ≈ 5.7%), driven in part by the Roger-Silva (32) resistance-training trial (NCT02598739) (32), which appeared in six reviews. The later Silva (33) active-comparator trial was treated as independent because it had a different registration (NCT04304326), comparator, and recruitment period (33). The observational psoriasis-prevalence review shared no primary studies with the intervention reviews. These repeated trial reports were treated as single underlying evidence bodies when interpreting effects.

**Table 5:** Primary-study overlap among the included reviews, by exercise/activity cluster.

Exercise/activity cluster	Contributing reviews	Reviews (*n*)	Unique studies	Inclusions	CCA	Overlap
Home-based exercise	R02	1	4	4	n/a	Not computable
Lifestyle/passive physical-therapy boundary	R02, R03, R04, R05	4	13	14	0.025641	Slight
Other exercise or rehabilitation	R01, R02, R04, R06	4	53	59	0.037736	Slight
Cluster A—HIIT/cardiorespiratory training	R01, R03, R04, R05, R06, R07, R08	7	6	12	0.166667	Very high
Cluster B—resistance/strength training	R01, R02, R03, R04, R05, R06, R07	7	111	149	0.057057	Moderate
Cluster C—fatigue/non-pharmacological PA behavior	R01, R02, R06, R07	4	74	84	0.045045	Slight
Cluster D—PA level/physical-fitness descriptive	R04, R05, R06, R09	4	50	56	0.040000	Slight
Overall (all included reviews)	R01, R02, R03, R04, R05, R06, R07, R08, R09	9	311	378	0.026929	Slight

Overlap was quantified with the corrected covered area (CCA) from the study-by-review citation grid. Thresholds were 0%–5% slight, >5%–10% moderate, >10%–15% high, and >15% very high. Overall overlap was slight (CCA ≈ 2.7%). The HIIT/cardiorespiratory cluster was very high (CCA ≈ 16.7%) because four reports from the same parent trial (NCT02995460) (28–31) appeared across seven reviews; the resistance/strength cluster was moderate (CCA ≈ 5.7%), partly reflecting the Roger-Silva (32) trial (NCT02598739) (32) in six reviews. The Silva (33) active-comparator trial (NCT04304326) (33) was treated as independent. The observational psoriasis-prevalence review shared no primary studies with intervention reviews. “Inclusions” denotes filled study-by-review cells; “Unique studies” denotes de-duplicated studies. Full definitions and verification are in **Supplementary Table S5**.

CCA, corrected covered area; HIIT, high-intensity interval training; n/a, not applicable (single-review cluster).

**Figure 3:**
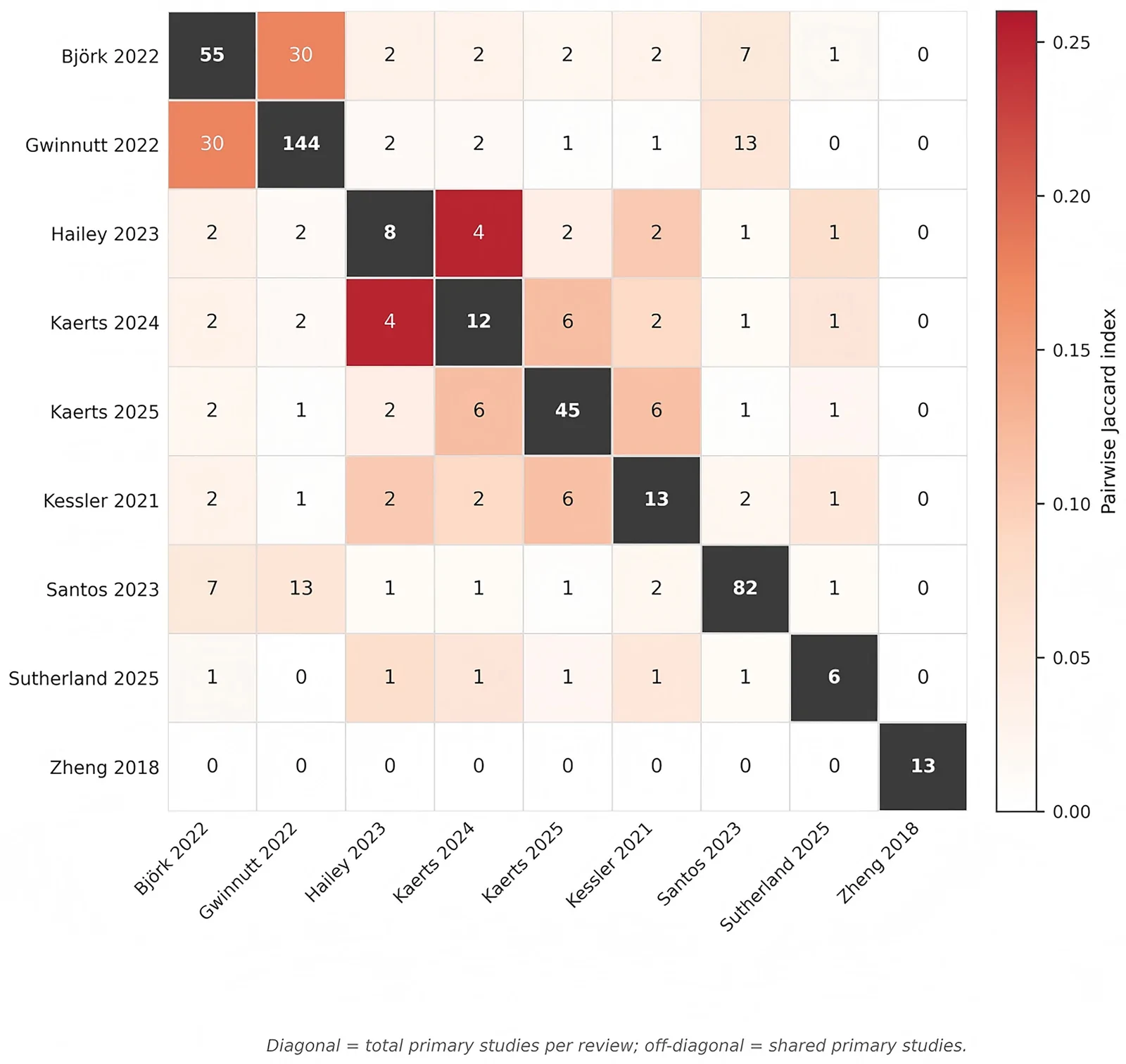
Primary-study overlap among the included reviews. Pairwise overlap in primary studies among the included reviews. Cell shading encodes the Jaccard index (shared studies divided by the union of studies for each pair); annotations give the number of shared primary studies, and diagonal values give each review’s own de-duplicated study count. Overlap was summarized with the corrected covered area (CCA), defined as (*N* − *r*)/(*r* × *c* − *r*), where *N* is the number of inclusions, *r* is the number of unique studies, and *c* is the number of reviews. Interpretation thresholds: 0%–5% slight, >5%–10% moderate, >10%–15% high, and >15% very high. Overall overlap across all nine reviews was slight (CCA ≈ 2.7%); overlap was concentrated in the high-intensity interval-training/cardiorespiratory cluster (CCA ≈ 16.7%, very high) and was moderate for the resistance/strength cluster (CCA ≈ 5.7%). The observational psoriasis-prevalence association review (R09) shared no primary studies with the intervention reviews. CCA, corrected covered area; PsA, psoriatic arthritis.

### Evidence landscape and certainty

The distribution of evidence across outcome domains and reviews is depicted in **Figure 4**, and certainty by domain is summarized in **Table 4**, with supporting detail in **Supplementary Tables S2**, **S4**. **Figure 5** displays the observational exposure–outcome association estimates. For intervention outcomes, the most conservative certainty was Very Low in every domain; only one cardiorespiratory-fitness construct reached Moderate certainty. For observational psoriasis-prevalence associations, the highest credibility was class III (suggestive); most associations reached no formal class because estimates were not statistically significant or required bias inputs were unavailable.

**Figure 4:**
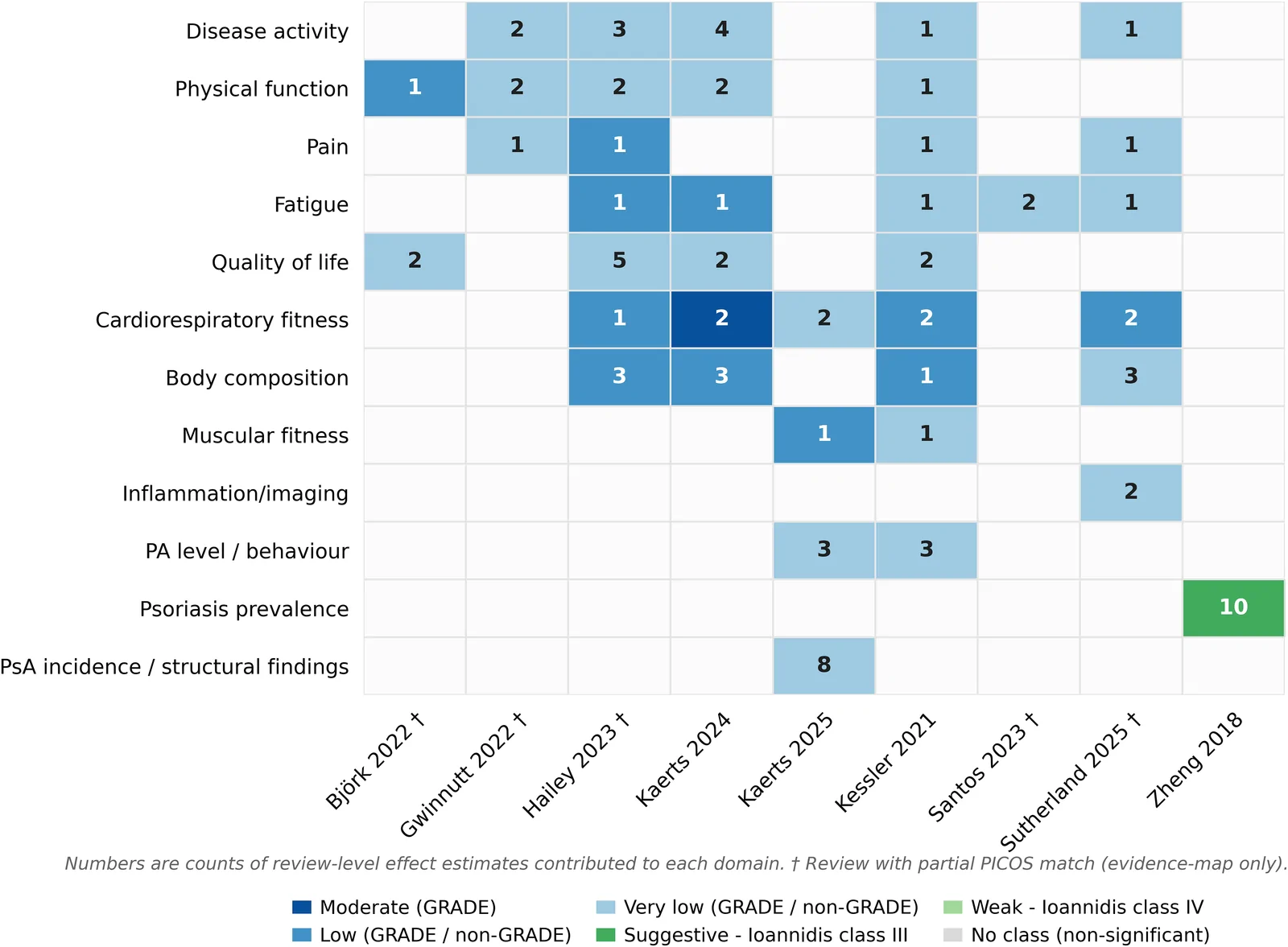
Evidence landscape across outcome domains and included reviews. Each filled cell indicates that a review (column) contributes evidence to an outcome domain (row); the number is the count of review-level effect estimates contributed. Cell color encodes the most conservative certainty or credibility across that review’s constructs in the domain: darker blue = higher GRADE certainty (Moderate → Low → Very low); green = Ioannidis class for the observational psoriasis-prevalence domain (suggestive class III versus weak class IV); pale gray = no class (non-significant association). Daggers (†) mark reviews with only a partial match to the umbrella-review PICOS, which are used for the evidence map rather than the main conclusions. GRADE, Grading of Recommendations Assessment, Development and Evaluation; PA, physical activity; PsA, psoriatic arthritis.

**Figure 5:**
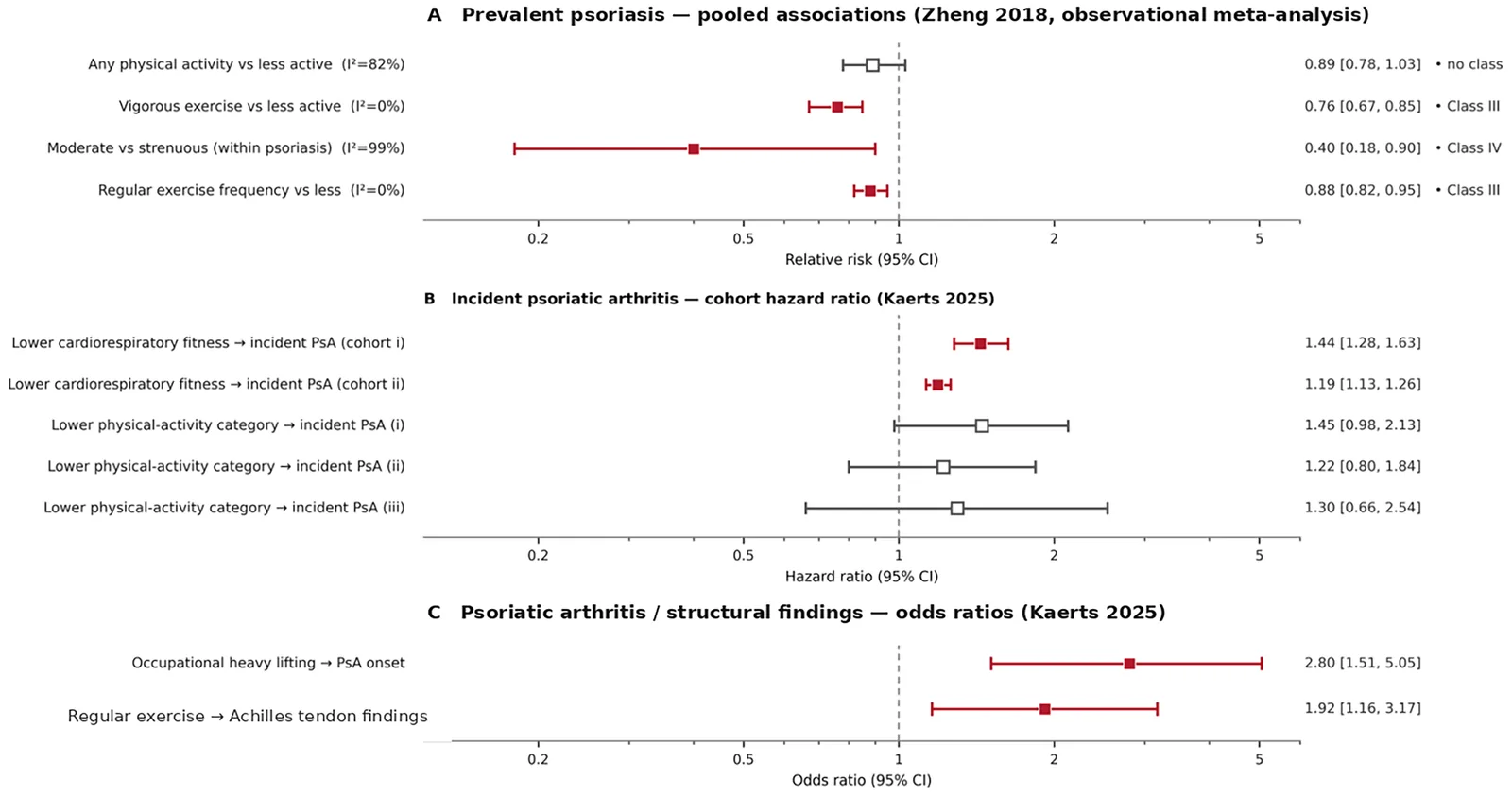
Observational exposure–outcome association estimates for physical activity and fitness. Review-reported observational association estimates with 95% confidence intervals, plotted on a logarithmic scale. **(A)** Pooled RRs for prevalent psoriasis from Zheng (26), annotated with Ioannidis credibility classes. **(B)** Hazard ratios for incident PsA from cohort studies reported by Kaerts (22). **(C)** Odds ratios for PsA onset and structural findings reported by Kaerts (22). Filled red markers denote estimates whose 95% confidence intervals exclude the null; hollow gray markers denote non-significant estimates. The vertical dashed line marks the null (ratio = 1). Estimates are presented as reported by each review; no umbrella-level pooling was performed, and the observational estimates do not establish causality. CI, confidence interval; HR, hazard ratio; OR, odds ratio; PsA, psoriatic arthritis; RR, relative risk/ratio as reported by the source review.

## Discussion

### Principal findings by disease and evidence type

This umbrella review yields different conclusions for the two diseases and evidence types. For cutaneous psoriasis, evidence was limited to one observational meta-analysis of prevalence; no eligible review isolated exercise effects on PASI, DLQI, or other skin outcomes. For PsA, several reviews existed, but the intervention evidence was concentrated in a small number of trials repeatedly reported across reviews. Overall confidence was predominantly Low or Critically Low, and the most conservative certainty was Very Low across intervention domains. The apparent volume of review literature therefore exceeds the amount of independent disease-specific evidence.

### Cutaneous psoriasis

For cutaneous psoriasis, the associations with vigorous activity and regular exercise frequency are biologically plausible in view of cardiometabolic and inflammatory pathways (3–6), but reverse causation and residual confounding remain credible: more severe disease, obesity, socioeconomic factors, or comorbidity may influence both activity and psoriasis prevalence. The non-significant overall estimate, high heterogeneity in several contrasts, and class III ceiling require cautious interpretation. These data support an association, not a causal preventive or therapeutic effect.

Broader lifestyle evidence is relevant context but does not resolve the exercise-specific question. In a 20-week randomized trial of 303 overweight or obese participants with psoriasis, Naldi et al. compared dietary restriction plus advice to undertake continuous aerobic activity (such as walking for at least 40 min three times weekly) with information alone; median PASI reduction was greater in the combined program (48.0% versus 25.5%) (37). A critically appraised synthesis of six randomized trials likewise found that lifestyle-induced weight loss improved PASI (mean change −2.59, 95% CI −4.09 to −1.09) (38). These findings support a plausible indirect pathway through weight reduction and metabolic health, but the multicomponent interventions do not isolate the effect of physical activity. They were therefore discussed as contextual evidence rather than included as exercise-effect estimates.

### Psoriatic arthritis

For PsA, HIIT and resistance exercise produced potentially meaningful changes in fitness, pain, disease activity, fatigue, function, and body composition, but almost all quantitative signals came from the Thomsen/Chronaiou or Roger-Silva trial families (28–32). The VO_2_max increase of 3.08–3.72 mL/kg/min was statistically significant, but its clinical importance is uncertain because no PsA-specific minimal important difference was identified. Pain and BASDAI point estimates exceeded available reference thresholds, the HAQ estimate was threshold-dependent, and fatigue remained statistically inconclusive. These results are best viewed as promising proof-of-concept signals rather than confirmed treatment effects.

### Why an umbrella review remains informative

The umbrella-review design remains informative despite the limited psoriasis evidence. For PsA, it reveals that several apparently concordant reviews rely on the same small trials and therefore should not be interpreted as independent confirmation. For cutaneous psoriasis, it demonstrates a more fundamental evidence gap: an observational prevalence literature and multicomponent weight-loss trials exist, but no eligible exercise-only systematic review establishes effects on skin disease. The overview therefore supports evidence mapping, quality appraisal, and duplication control, but not an umbrella-level pooled effect or a mature disease-specific prescription.

### Strengths and limitations

Strengths include the six-database search, independent duplicate screening and extraction, explicit separation of intervention and observational evidence, AMSTAR 2 appraisal, identification of rating provenance, and primary-study overlap analysis. Limitations are substantial. Only nine reviews were eligible, only one addressed cutaneous psoriasis directly and it was observational, and no exercise-only review evaluated skin severity. PsA estimates were based mainly on small repeated trials and often lacked confidence intervals or responder analyses. Some certainty ratings were necessarily applied *de novo* from incomplete review-level reporting. No umbrella-level pooling was valid. Although adverse events, flares, and safety outcomes were prespecified, review-level reporting was sparse, inconsistently defined, and often absent; only a narrative summary was possible, and absence of reported harm must not be interpreted as evidence of safety.

### Implications for practice and research

General physical-activity recommendations remain relevant for overall and cardiometabolic health (6, 7), but this review does not establish a disease-specific exercise prescription or safety profile for psoriasis or PsA. Future cutaneous-psoriasis trials should isolate exercise from dietary weight loss and assess PASI, symptoms, quality of life, cardiometabolic mediators, and adverse events. PsA trials should be adequately powered and prospectively registered, use PsA-specific disease-activity and function measures, prespecify clinically important responder thresholds, report adherence and harms, and compare reproducible exercise doses. Longitudinal studies should distinguish leisure activity from occupational mechanical loading when evaluating PsA onset and structural progression. Future reviews should provide excluded-study lists, register protocols prospectively, and avoid counting multiple reports of the same trial as independent evidence.

## Conclusion

For cutaneous psoriasis, vigorous activity and regular exercise frequency were associated with lower prevalence, but the evidence was observational and does not establish prevention or improvement of skin disease. For PsA, small HIIT and resistance-training trials reported possible improvements in fitness and selected clinical outcomes, but the evidence was repeatedly represented across reviews and remained of Very Low certainty. Safety reporting was incomplete and did not permit comparative risk estimates or a disease-specific conclusion. General physical-activity recommendations may still be followed for established overall-health indications, but disease-specific efficacy, dose, and safety conclusions for psoriasis or PsA cannot yet be made confidently.
