# Supplementary material for: Exercise, physical activity, and physical fitness in psoriasis and psoriatic arthritis: an umbrella review
Source: Front Immunol. 2026 Oct 9;17:1921728. doi: 10.3389/fimmu.2026.1921728 (PMC13690634; doi:10.3389/fimmu.2026.1921728)
Supplement: Supplementary file 1 [file DataSheet1.docx]

**Supplementary Materials**

Supplementary tables provide the complete review-level evidence underlying the streamlined main manuscript. Table S2 retains every extracted estimate, including direction-only, NR, duplicate-review, and evidence-map-only rows removed from the simplified main Table 2. Table S4 identifies certainty-rating provenance, Table S5 verifies the corrected-covered-area calculations, Table S6 summarises exercise programmes and trial identity, Table S7 documents review-by-review evidence-tier decisions, and Table S8 summarises safety and adverse-event reporting.

**Table S1. Database search strategies**

Six databases were searched from inception to March 1, 2026, reported in line with PRISMA-S. Concept blocks combine the population (psoriasis / psoriatic arthritis) with the exercise / physical-activity / physical-fitness exposure and a systematic-review / meta-analysis design filter. The strings below reflect the search as executed; controlled-vocabulary terms (MeSH, Emtree, subject headings) are exploded where indicated.

**PubMed**

Searched from inception to March 1, 2026. No language restriction applied at the search stage.

| **Set** | **Query** |
| --- | --- |
| #1 | "Psoriasis"[Mesh] OR "Arthritis, Psoriatic"[Mesh] |
| #2 | psoriasis[tiab] OR psoriatic[tiab] OR "psoriatic arthritis"[tiab] |
| #3 | #1 OR #2 |
| #4 | "Exercise"[Mesh] OR "Exercise Therapy"[Mesh] OR "Physical Fitness"[Mesh] OR "High-Intensity Interval Training"[Mesh] OR "Resistance Training"[Mesh] OR "Walking"[Mesh] OR "Cardiorespiratory Fitness"[Mesh] |
| #5 | exercise[tiab] OR "physical activity"[tiab] OR "physical fitness"[tiab] OR "physical training"[tiab] OR "aerobic training"[tiab] OR "resistance training"[tiab] OR "strength training"[tiab] OR "endurance training"[tiab] OR HIIT[tiab] OR "high-intensity interval training"[tiab] OR "cardiorespiratory fitness"[tiab] OR walking[tiab] |
| #6 | #4 OR #5 |
| #7 | "Systematic Review"[Publication Type] OR "Meta-Analysis"[Publication Type] OR "Systematic Review"[Mesh] OR "Meta-Analysis as Topic"[Mesh] |
| #8 | "systematic review"[tiab] OR "meta-analysis"[tiab] OR "meta analysis"[tiab] OR "umbrella review"[tiab] OR "overview of reviews"[tiab] |
| #9 | #7 OR #8 |
| #10 | #3 AND #6 AND #9 |
| #11 | #10 with date limit: ("1900/01/01"[Date - Publication] : "2026/03/01"[Date - Publication]) |

**Embase**

Searched from inception to March 1, 2026. Emtree explosion ('/exp') applied to subject headings.

| **Set** | **Query** |
| --- | --- |
| #1 | 'psoriasis'/exp OR 'psoriatic arthritis'/exp |
| #2 | (psoriasis OR psoriatic OR 'psoriatic arthritis'):ti,ab |
| #3 | #1 OR #2 |
| #4 | 'exercise'/exp OR 'fitness'/exp OR 'kinesiotherapy'/exp OR 'high intensity interval training'/exp OR 'resistance training'/exp OR 'aerobic exercise'/exp OR 'walking'/exp |
| #5 | (exercise OR 'physical activity' OR 'physical fitness' OR 'physical training' OR 'aerobic training' OR 'resistance training' OR 'strength training' OR 'endurance training' OR HIIT OR 'cardiorespiratory fitness' OR walking):ti,ab |
| #6 | #4 OR #5 |
| #7 | 'systematic review'/exp OR 'meta analysis'/exp OR 'meta analysis (topic)'/exp |
| #8 | ('systematic review' OR 'meta-analysis' OR 'meta analysis' OR 'umbrella review' OR 'overview of reviews'):ti,ab |
| #9 | #7 OR #8 |
| #10 | #3 AND #6 AND #9 |
| #11 | #10 AND [1900-2026]/py (records to 1 March 2026) |

**Cochrane Library**

Searched from inception to March 1, 2026. MeSH headings searched with explosion; the systematic-review filter is applied by limiting to Cochrane Reviews and review records.

| **Set** | **Query** |
| --- | --- |
| #1 | MeSH descriptor: [Psoriasis] explode all trees |
| #2 | MeSH descriptor: [Arthritis, Psoriatic] explode all trees |
| #3 | (psoriasis OR psoriatic):ti,ab,kw |
| #4 | #1 OR #2 OR #3 |
| #5 | MeSH descriptor: [Exercise] explode all trees |
| #6 | MeSH descriptor: [Physical Fitness] explode all trees |
| #7 | (exercise OR "physical activity" OR "physical fitness" OR "resistance training" OR "strength training" OR "aerobic training" OR HIIT OR "high-intensity interval training" OR "cardiorespiratory fitness" OR walking):ti,ab,kw |
| #8 | #5 OR #6 OR #7 |
| #9 | #4 AND #8 |
| #10 | #9 limited to Cochrane Reviews / review records, publication date to 1 March 2026 |

**Web of Science**

Searched from inception to March 1, 2026. Topic field (TS) searches title, abstract, author keywords, and Keywords Plus; design filter applied through the document-type / topic review terms.

| **Set** | **Query** |
| --- | --- |
| #1 | TS=(psoriasis OR psoriatic OR "psoriatic arthritis") |
| #2 | TS=(exercise OR "physical activity" OR "physical fitness" OR "physical training" OR "aerobic training" OR "resistance training" OR "strength training" OR "endurance training" OR HIIT OR "high-intensity interval training" OR "cardiorespiratory fitness" OR walking) |
| #3 | TS=("systematic review" OR "meta-analysis" OR "meta analysis" OR "umbrella review" OR "overview of reviews") |
| #4 | #1 AND #2 AND #3 |
| #5 | #4, refined by publication years up to 2026 (records to 1 March 2026) |

**Scopus**

Searched from inception to March 1, 2026. TITLE-ABS-KEY searches title, abstract, and keywords.

| **Set** | **Query** |
| --- | --- |
| #1 | TITLE-ABS-KEY ( psoriasis OR psoriatic OR "psoriatic arthritis" ) |
| #2 | TITLE-ABS-KEY ( exercise OR "physical activity" OR "physical fitness" OR "physical training" OR "aerobic training" OR "resistance training" OR "strength training" OR "endurance training" OR HIIT OR "high-intensity interval training" OR "cardiorespiratory fitness" OR walking ) |
| #3 | TITLE-ABS-KEY ( "systematic review" OR "meta-analysis" OR "meta analysis" OR "umbrella review" OR "overview of reviews" ) |
| #4 | #1 AND #2 AND #3 |
| #5 | #4 AND PUBYEAR < 2027 (limited to records published up to 1 March 2026) |

**SPORTDiscus (EBSCOhost)**

Searched from inception to March 1, 2026; selected as the sixth, topic-specific database for this exercise-focused review. Field codes: TI = title, AB = abstract, SU = subject terms.

| **Set** | **Query** |
| --- | --- |
| #1 | TI ( psoriasis OR psoriatic ) OR AB ( psoriasis OR psoriatic ) OR SU ( psoriasis OR psoriatic arthritis ) |
| #2 | TI ( exercise OR "physical activity" OR "physical fitness" OR "resistance training" OR "strength training" OR "aerobic training" OR HIIT OR "cardiorespiratory fitness" OR walking ) OR AB ( exercise OR "physical activity" OR "physical fitness" OR "resistance training" OR "strength training" OR "aerobic training" OR HIIT OR "cardiorespiratory fitness" OR walking ) |
| #3 | TI ( "systematic review" OR "meta-analysis" OR "meta analysis" OR "umbrella review" ) OR AB ( "systematic review" OR "meta-analysis" OR "meta analysis" OR "umbrella review" ) |
| #4 | #1 AND #2 AND #3 |
| #5 | #4, limited to publication dates up to 1 March 2026 |

Abbreviations: AB, abstract field; tiab / TS / TITLE-ABS-KEY, title–abstract(–keyword) fields in the respective interfaces; MeSH, Medical Subject Headings; /exp, Emtree explosion; SU, subject-heading field; TI, title field.

**Table S2. Outcome-level evidence: all extracted review-level effect estimates**

| **Effect ID** | **Review** | **Domain** | **Standardized construct** | **Comparator** | **Metric** | **Estimate (95% CI)** | **I² / k** | **Evidence tier** | **Notes / exclusion reason** |
| --- | --- | --- | --- | --- | --- | --- | --- | --- | --- |
| R01-E01 | R01 | Quality of life | Quality of life - SF-36 physical component | Inactive/no-exercise control | MD | 12.05 (-3.25 to 27.35) | I² NR k 1 | Evidence-map-only · map only | Partial PICOS match; contributes to evidence map only, not main conclusions. — Analysis type: Meta-analysis subgroup / forest plot. Native-scale estimate carried as reported; no cross-review pooling (overlap and metric heterogeneity). Source label: PsA subgroup. |
| R01-E02 | R01 | Quality of life | Quality of life - SF-36 mental component | Inactive/no-exercise control | MD | 5.90 (-9.05 to 20.85) | I² NR k 1 | Evidence-map-only · map only | Partial PICOS match; contributes to evidence map only, not main conclusions. — Analysis type: Meta-analysis subgroup / forest plot. Native-scale estimate carried as reported; no cross-review pooling (overlap and metric heterogeneity). Source label: PsA subgroup. |
| R01-E03 | R01 | Physical function | Physical function - activity performance | Inactive/no-exercise control | SMD | -0.57 (-1.01 to -0.13) | I² 0% k 1 | Evidence-map-only · map only | Partial PICOS match; contributes to evidence map only, not main conclusions. — Analysis type: Meta-analysis subgroup / narrative result. Native-scale estimate carried as reported; no cross-review pooling (overlap and metric heterogeneity). Source label: PsA subgroup. |
| R02-E01 | R02 | Physical function | Functional capacity - BASFI | Wait-list/usual-care control | SMD | -0.50 (-1.12 to 0.12) | I² NR k 1 | Evidence-map-only · map only | Partial PICOS match; contributes to evidence map only, not main conclusions. — Analysis type: Original-study evidence table within systematic review. Native-scale estimate carried as reported; no cross-review pooling (overlap and metric heterogeneity). Source label: Roger-Silva resistance-training RCT. |
| R02-E02 | R02 | Physical function | Physical function - HAQ | Wait-list/usual-care control | SMD | -0.65 (-1.28 to -0.02) | I² NR k 1 | Evidence-map-only · map only | Partial PICOS match; contributes to evidence map only, not main conclusions. — Analysis type: Original-study evidence table within systematic review. Native-scale estimate carried as reported; no cross-review pooling (overlap and metric heterogeneity). Source label: Roger-Silva resistance-training RCT. |
| R02-E03 | R02 | Disease activity | Disease activity - BASDAI | Wait-list/usual-care control | SMD | -0.66 (-1.29 to -0.03) | I² NR k 1 | Evidence-map-only · map only | Partial PICOS match; contributes to evidence map only, not main conclusions. — Analysis type: Original-study evidence table within systematic review. Native-scale estimate carried as reported; no cross-review pooling (overlap and metric heterogeneity). Source label: Roger-Silva resistance-training RCT. |
| R02-E04 | R02 | Disease activity | Disease activity - DAS28 | Wait-list/usual-care control | SMD | -0.42 (-1.04 to 0.20) | I² NR k 1 | Evidence-map-only · map only | Partial PICOS match; contributes to evidence map only, not main conclusions. — Analysis type: Original-study evidence table within systematic review. Native-scale estimate carried as reported; no cross-review pooling (overlap and metric heterogeneity). Source label: Roger-Silva resistance-training RCT. |
| R02-E05 | R02 | Pain | Pain - VAS | No comparator/pre-post only | Descriptive | Baseline 43.7; 4 weeks 34.0; 12 weeks 48.6 | I² NR k 1 | Evidence-map-only · map only | Partial PICOS match; contributes to evidence map only, not main conclusions. — Analysis type: Single-arm descriptive evidence table. Point estimate without computable 95% CI as reported. Native-scale estimate carried as reported; no cross-review pooling (overlap and metric heterogeneity). Source label: Chimenti home exercise single-arm study. |
| R03-E01 | R03 | Physical function | Physical function - HAQ | Wait-list/usual-care control | Narrative | NR | I² NR k 1 | Evidence-map-only · map only | Partial PICOS match; contributes to evidence map only, not main conclusions. — Analysis type: Narrative synthesis. Native-scale estimate carried as reported; no cross-review pooling (overlap and metric heterogeneity). Source label: Roger-Silva resistance-training RCT. |
| R03-E02 | R03 | Disease activity | Disease activity - BASDAI | Wait-list/usual-care control | Narrative | NR | I² NR k 1 | Evidence-map-only · map only | Partial PICOS match; contributes to evidence map only, not main conclusions. — Analysis type: Narrative synthesis. Native-scale estimate carried as reported; no cross-review pooling (overlap and metric heterogeneity). Source label: Roger-Silva resistance-training RCT. |
| R03-E03 | R03 | Disease activity | Disease activity - patient global assessment | No-change/usual-care control | MD | -0.49 (-10.91 to 9.94) | I² NR k 1 | Evidence-map-only · map only | Partial PICOS match; contributes to evidence map only, not main conclusions. — Analysis type: Narrative synthesis with extracted mean difference. Native-scale estimate carried as reported; no cross-review pooling (overlap and metric heterogeneity). Source label: Thomsen HIIT PsA RCT. |
| R03-E04 | R03 | Disease activity | Disease activity - DAS44 | No-change/usual-care control | MD | -0.08 (-0.36 to 0.20) | I² NR k 1 | Evidence-map-only · map only | Partial PICOS match; contributes to evidence map only, not main conclusions. — Analysis type: Narrative synthesis with extracted mean difference. Native-scale estimate carried as reported; no cross-review pooling (overlap and metric heterogeneity). Source label: Thomsen HIIT PsA RCT. |
| R03-E05 | R03 | Pain | Pain - VAS | No-change/usual-care control | MD | 5.45 (-4.36 to 15.26) | I² NR k 1 | Evidence-map-only · map only | Partial PICOS match; contributes to evidence map only, not main conclusions. — Analysis type: Narrative synthesis with extracted mean difference. Native-scale estimate carried as reported; no cross-review pooling (overlap and metric heterogeneity). Source label: Thomsen HIIT PsA RCT. |
| R03-E06 | R03 | Fatigue | Fatigue - VAS | No-change/usual-care control | MD | -12.83 (-25.88 to 0.23) | I² NR k 1 | Evidence-map-only · map only | Partial PICOS match; contributes to evidence map only, not main conclusions. — Analysis type: Narrative synthesis with extracted mean difference. Native-scale estimate carried as reported; no cross-review pooling (overlap and metric heterogeneity). Source label: Thomsen HIIT PsA RCT. |
| R03-E07 | R03 | Cardiorespiratory fitness | Cardiorespiratory fitness - VO2max | No-change/usual-care control | MD | 3.72 (2.38 to 5.06) | I² NR k 1 | Evidence-map-only · map only | Partial PICOS match; contributes to evidence map only, not main conclusions. — Analysis type: Narrative synthesis with extracted mean difference. Native-scale estimate carried as reported; no cross-review pooling (overlap and metric heterogeneity). Source label: Thomsen HIIT PsA RCT. |
| R03-E08 | R03 | Body composition | Body composition - truncal fat percentage | No-change/usual-care control | MD | -1.28 (-2.51 to -0.05) | I² NR k 1 | Evidence-map-only · map only | Partial PICOS match; contributes to evidence map only, not main conclusions. — Analysis type: Narrative synthesis with extracted mean difference. Native-scale estimate carried as reported; no cross-review pooling (overlap and metric heterogeneity). Source label: Thomsen HIIT PsA RCT. |
| R03-E09 | R03 | Body composition | Body composition - total body fat percentage | No-change/usual-care control | MD | -0.80 (-1.71 to 0.10) | I² NR k 1 | Evidence-map-only · map only | Partial PICOS match; contributes to evidence map only, not main conclusions. — Analysis type: Narrative synthesis with extracted mean difference. Native-scale estimate carried as reported; no cross-review pooling (overlap and metric heterogeneity). Source label: Thomsen HIIT PsA RCT. |
| R03-E10 | R03 | Body composition | Body mass index | No-change/usual-care control | MD | -0.31 (-0.78 to 0.17) | I² NR k 1 | Evidence-map-only · map only | Partial PICOS match; contributes to evidence map only, not main conclusions. — Analysis type: Narrative synthesis with extracted mean difference. Native-scale estimate carried as reported; no cross-review pooling (overlap and metric heterogeneity). Source label: Thomsen HIIT PsA RCT. |
| R03-E11 | R03 | Quality of life | Pain-related quality of life - SF-36 bodily pain | No comparator/correlation only | r | -0.30 | I² NR k 1 | Evidence-map-only · map only | Partial PICOS match; contributes to evidence map only, not main conclusions. — Analysis type: Nonrandomized correlation evidence. Point estimate without computable 95% CI as reported. Native-scale estimate carried as reported; no cross-review pooling (overlap and metric heterogeneity). Source label: Chimenti home exercise nonrandomized study. |
| R03-E12 | R03 | Physical function | Physical function - HAQ | No comparator/correlation only | r | 0.24 | I² NR k 1 | Evidence-map-only · map only | Partial PICOS match; contributes to evidence map only, not main conclusions. — Analysis type: Nonrandomized correlation evidence. Point estimate without computable 95% CI as reported. Native-scale estimate carried as reported; no cross-review pooling (overlap and metric heterogeneity). Source label: Chimenti home exercise nonrandomized study. |
| R03-E13 | R03 | Quality of life | Quality of life - SF-36 role physical | No comparator/correlation only | r | 0.23 | I² NR k 1 | Evidence-map-only · map only | Partial PICOS match; contributes to evidence map only, not main conclusions. — Analysis type: Nonrandomized correlation evidence. Point estimate without computable 95% CI as reported. Native-scale estimate carried as reported; no cross-review pooling (overlap and metric heterogeneity). Source label: Chimenti home exercise nonrandomized study. |
| R03-E14 | R03 | Quality of life | Quality of life - SF-36 physical function | No comparator/correlation only | r | 0.28 | I² NR k 1 | Evidence-map-only · map only | Partial PICOS match; contributes to evidence map only, not main conclusions. — Analysis type: Nonrandomized correlation evidence. Point estimate without computable 95% CI as reported. Native-scale estimate carried as reported; no cross-review pooling (overlap and metric heterogeneity). Source label: Chimenti home exercise nonrandomized study. |
| R03-E15 | R03 | Quality of life | Quality of life - SF-36 vitality | No comparator/correlation only | r | 0.23 | I² NR k 1 | Evidence-map-only · map only | Partial PICOS match; contributes to evidence map only, not main conclusions. — Analysis type: Nonrandomized correlation evidence. Point estimate without computable 95% CI as reported. Native-scale estimate carried as reported; no cross-review pooling (overlap and metric heterogeneity). Source label: Chimenti home exercise nonrandomized study. |
| R03-E16 | R03 | Quality of life | Quality of life - SF-36 social functioning | No comparator/correlation only | r | -0.44 | I² NR k 1 | Evidence-map-only · map only | Partial PICOS match; contributes to evidence map only, not main conclusions. — Analysis type: Nonrandomized correlation evidence. Point estimate without computable 95% CI as reported. Native-scale estimate carried as reported; no cross-review pooling (overlap and metric heterogeneity). Source label: Chimenti home exercise nonrandomized study. |
| R04-E01 | R04 | Cardiorespiratory fitness | Cardiorespiratory fitness - VO2max | No-change/usual-care control | MD | 3.72 (2.38 to 5.06) | I² NR k 1 | Core-eligible · in primary | Analysis type: Narrative synthesis with extracted trial estimate. Native-scale estimate carried as reported; no cross-review pooling (overlap and metric heterogeneity). Source label: Thomsen HIIT PsA RCT. |
| R04-E02 | R04 | Cardiorespiratory fitness | Cardiorespiratory fitness - VO2max | No-change/usual-care control | MD | 3.08 (1.63 to 4.53) | I² NR k 1 | Core-eligible · in primary | Analysis type: Narrative synthesis with extracted trial estimate. Native-scale estimate carried as reported; no cross-review pooling (overlap and metric heterogeneity). Source label: Thomsen HIIT PsA RCT. |
| R04-E03 | R04 | Body composition | Body composition - truncal fat percentage | No-change/usual-care control | MD | -1.28 (-2.51 to -0.05) | I² NR k 1 | Core-eligible · in primary | Analysis type: Narrative synthesis with extracted trial estimate. Native-scale estimate carried as reported; no cross-review pooling (overlap and metric heterogeneity). Source label: Thomsen HIIT PsA RCT. |
| R04-E04 | R04 | Body composition | Body composition - total body fat percentage | No-change/usual-care control | MD | -0.80 (-1.71 to 0.10) | I² NR k 1 | Core-eligible · in primary | Analysis type: Narrative synthesis with extracted trial estimate. Native-scale estimate carried as reported; no cross-review pooling (overlap and metric heterogeneity). Source label: Thomsen HIIT PsA RCT. |
| R04-E05 | R04 | Body composition | Body mass index | No-change/usual-care control | MD | -0.31 (-0.78 to 0.17) | I² NR k 1 | Core-eligible · in primary | Analysis type: Narrative synthesis with extracted trial estimate. Native-scale estimate carried as reported; no cross-review pooling (overlap and metric heterogeneity). Source label: Thomsen HIIT PsA RCT. |
| R04-E06 | R04 | Fatigue | Fatigue - VAS | No-change/usual-care control | MD | -12.83 (-25.88 to 0.23) | I² NR k 1 | Core-eligible · in primary | Analysis type: Narrative synthesis with extracted trial estimate. Native-scale estimate carried as reported; no cross-review pooling (overlap and metric heterogeneity). Source label: Thomsen HIIT PsA RCT. |
| R04-E07 | R04 | Disease activity | Disease activity - composite disease activity | No-change/usual-care control | Narrative | NR | I² NR k 1 | Core-eligible · in primary | Analysis type: Narrative synthesis. Native-scale estimate carried as reported; no cross-review pooling (overlap and metric heterogeneity). Source label: Thomsen HIIT PsA RCT. |
| R04-E08 | R04 | Disease activity | Disease activity - BASDAI | Wait-list/usual-care control | Direction only (estimate NR) | NR | I² NR k 1 | Core-eligible · in primary | Analysis type: Narrative synthesis with reported p-value. Native-scale estimate carried as reported; no cross-review pooling (overlap and metric heterogeneity). Source label: Roger-Silva resistance-training RCT. |
| R04-E09 | R04 | Physical function | Physical function - HAQ | Wait-list/usual-care control | Direction only (estimate NR) | NR | I² NR k 1 | Core-eligible · in primary | Analysis type: Narrative synthesis with reported p-value. Native-scale estimate carried as reported; no cross-review pooling (overlap and metric heterogeneity). Source label: Roger-Silva resistance-training RCT. |
| R04-E10 | R04 | Quality of life | Pain-related quality of life - SF-36 bodily pain | Wait-list/usual-care control | Direction only (estimate NR) | NR | I² NR k 1 | Core-eligible · in primary | Analysis type: Narrative synthesis with reported p-value. Native-scale estimate carried as reported; no cross-review pooling (overlap and metric heterogeneity). Source label: Roger-Silva resistance-training RCT. |
| R04-E11 | R04 | Quality of life | Quality of life - SF-36 general health | Wait-list/usual-care control | Direction only (estimate NR) | NR | I² NR k 1 | Core-eligible · in primary | Analysis type: Narrative synthesis with reported p-value. Native-scale estimate carried as reported; no cross-review pooling (overlap and metric heterogeneity). Source label: Roger-Silva resistance-training RCT. |
| R04-E12 | R04 | Physical function | Functional capacity - BASFI | Wait-list/usual-care control | Direction only (estimate NR) | NR | I² NR k 1 | Core-eligible · in primary | Analysis type: Narrative synthesis with reported p-value. Native-scale estimate carried as reported; no cross-review pooling (overlap and metric heterogeneity). Source label: Roger-Silva resistance-training RCT. |
| R04-E13 | R04 | Disease activity | Disease activity - DAS28 | Wait-list/usual-care control | Direction only (estimate NR) | NR | I² NR k 1 | Core-eligible · in primary | Analysis type: Narrative synthesis with reported p-value. Native-scale estimate carried as reported; no cross-review pooling (overlap and metric heterogeneity). Source label: Roger-Silva resistance-training RCT. |
| R04-E14 | R04 | Disease activity | Composite clinical outcomes | Active exercise comparator | Narrative | NR | I² NR k 1 | Core-eligible · in primary | Analysis type: Narrative synthesis. Native-scale estimate carried as reported; no cross-review pooling (overlap and metric heterogeneity). Source label: Silva 2023 active-comparator RCT. |
| R05-E01 | R05 | Cardiorespiratory fitness | Cardiorespiratory fitness - VO2peak | General-population/reference comparator | Descriptive (mean, SD) | 29.76 (7.30) | I² NR k 1 | Core-eligible · in primary | Analysis type: Descriptive synthesis. Point estimate without computable 95% CI as reported. Native-scale estimate carried as reported; no cross-review pooling (overlap and metric heterogeneity). Source label: Physical fitness descriptive evidence. |
| R05-E02 | R05 | Cardiorespiratory fitness | Cardiorespiratory fitness - VO2peak | Healthy/control comparator | Descriptive (median, IQR) | 19.5 (15.8 to 22.9) | I² NR k 1 | Core-eligible · in primary | Analysis type: Descriptive synthesis. Point estimate without computable 95% CI as reported. Native-scale estimate carried as reported; no cross-review pooling (overlap and metric heterogeneity). Source label: Obese PsA vs obese controls. |
| R05-E03 | R05 | PsA incidence / structural associations | Psoriatic arthritis risk/incidence | High-fitness/high-activity reference | HR | 1.44 (1.28 to 1.63) | I² NR k 1 | Core-eligible · in primary | Analysis type: Prospective cohort association summarized in systematic review. Native-scale estimate carried as reported; no cross-review pooling (overlap and metric heterogeneity). Source label: CRF and incident PsA cohort. |
| R05-E04 | R05 | PsA incidence / structural associations | Psoriatic arthritis risk/incidence | High-fitness/high-activity reference | HR | 1.19 (1.13 to 1.26) | I² NR k 1 | Core-eligible · in primary | Analysis type: Prospective cohort association summarized in systematic review. Native-scale estimate carried as reported; no cross-review pooling (overlap and metric heterogeneity). Source label: CRF and incident PsA cohort. |
| R05-E05 | R05 | Muscular fitness | Muscular fitness - handgrip strength | Healthy/control comparator | Descriptive | Range 11.33-56.70 kg and 231.92-285 mmHg across studies | I² NR k 7 | Core-eligible · in primary | Analysis type: Descriptive synthesis. Point estimate without computable 95% CI as reported. Native-scale estimate carried as reported; no cross-review pooling (overlap and metric heterogeneity). Source label: Handgrip strength evidence. |
| R05-E06 | R05 | Physical activity level/behaviour | Physical activity level - MVPA | Healthy/control comparator | Descriptive | 47 (35) to 90.5 (40.8) min/day across two accelerometry studies | I² NR k 2 | Core-eligible · in primary | Analysis type: Descriptive synthesis. Point estimate without computable 95% CI as reported. Native-scale estimate carried as reported; no cross-review pooling (overlap and metric heterogeneity). Source label: Objective PA evidence. |
| R05-E07 | R05 | Physical activity level/behaviour | Disease activity and daily mood association | Lower-fitness/lower-activity reference | Narrative | NR | I² NR k 2 | Core-eligible · in primary | Analysis type: Association synthesis. Native-scale estimate carried as reported; no cross-review pooling (overlap and metric heterogeneity). Source label: Objective PA and disease/mood association. |
| R05-E08 | R05 | Physical activity level/behaviour | Physical activity guideline attainment | Guideline/reference threshold | Descriptive | 17% to 69% meeting selected PA recommendations across studies | I² NR k Multiple studies | Core-eligible · in primary | Analysis type: Descriptive synthesis. Point estimate without computable 95% CI as reported. Native-scale estimate carried as reported; no cross-review pooling (overlap and metric heterogeneity). Source label: Subjective PA evidence. |
| R05-E09 | R05 | PsA incidence / structural associations | Psoriatic arthritis risk/incidence | High-fitness/high-activity reference | HR | 1.45 (0.98 to 2.13) | I² NR k 1 | Core-eligible · in primary | Analysis type: Prospective cohort association summarized in systematic review. Native-scale estimate carried as reported; no cross-review pooling (overlap and metric heterogeneity). Source label: PA level and incident PsA cohort. |
| R05-E10 | R05 | PsA incidence / structural associations | Psoriatic arthritis risk/incidence | High-fitness/high-activity reference | HR | 1.22 (0.80 to 1.84) | I² NR k 1 | Core-eligible · in primary | Analysis type: Prospective cohort association summarized in systematic review. Native-scale estimate carried as reported; no cross-review pooling (overlap and metric heterogeneity). Source label: PA level and incident PsA cohort. |
| R05-E11 | R05 | PsA incidence / structural associations | Psoriatic arthritis risk/incidence | High-fitness/high-activity reference | HR | 1.30 (0.66 to 2.54) | I² NR k 1 | Core-eligible · in primary | Analysis type: Prospective cohort association summarized in systematic review. Native-scale estimate carried as reported; no cross-review pooling (overlap and metric heterogeneity). Source label: PA level and incident PsA cohort. |
| R05-E12 | R05 | PsA incidence / structural associations | Psoriatic arthritis risk/incidence | Lower-fitness/lower-activity reference | OR | 2.80 (1.51 to 5.05) | I² NR k 1 | Core-eligible · in primary | Analysis type: Observational association synthesis. Native-scale estimate carried as reported; no cross-review pooling (overlap and metric heterogeneity). Source label: Biomechanical/PA association evidence. |
| R05-E13 | R05 | PsA incidence / structural associations | Structural damage - Achilles tendon | Lower-fitness/lower-activity reference | OR | 1.92 (1.16 to 3.17) | I² NR k 1 | Core-eligible · in primary | Analysis type: Observational association synthesis. Native-scale estimate carried as reported; no cross-review pooling (overlap and metric heterogeneity). Source label: Biomechanical/PA association evidence. |
| R05-E14 | R05 | PsA incidence / structural associations | Structural damage - peripheral radiographic damage | Lower-fitness/lower-activity reference | Beta | 0.16 | I² NR k 1 | Core-eligible · in primary | Analysis type: Observational association synthesis. Point estimate without computable 95% CI as reported. Native-scale estimate carried as reported; no cross-review pooling (overlap and metric heterogeneity). Source label: Biomechanical/PA association evidence. |
| R06-E01 | R06 | Physical activity level/behaviour | Physical activity guideline attainment | Guideline/reference threshold | Proportion | 68% | I² NR k 1 | Core-eligible · in primary | Analysis type: Descriptive synthesis. Point estimate without computable 95% CI as reported. Native-scale estimate carried as reported; no cross-review pooling (overlap and metric heterogeneity). Source label: PA level table. |
| R06-E02 | R06 | Physical activity level/behaviour | Physical activity guideline attainment | Guideline/reference threshold | Proportion | 17% | I² NR k 1 | Core-eligible · in primary | Analysis type: Descriptive synthesis. Point estimate without computable 95% CI as reported. Native-scale estimate carried as reported; no cross-review pooling (overlap and metric heterogeneity). Source label: PA level table. |
| R06-E03 | R06 | Physical activity level/behaviour | Physical activity participation | Guideline/reference threshold | Proportion | 48%; 64% of inactive avoided PA | I² NR k 1 | Core-eligible · in primary | Analysis type: Descriptive synthesis. Point estimate without computable 95% CI as reported. Native-scale estimate carried as reported; no cross-review pooling (overlap and metric heterogeneity). Source label: PA level table. |
| R06-E04 | R06 | Fatigue | Fatigue - VAS | No-change/usual-care control | MD | -12.83 (-25.88 to 0.23) | I² NR k 1 | Core-eligible · in primary | Analysis type: Narrative synthesis with extracted trial estimate. Native-scale estimate carried as reported; no cross-review pooling (overlap and metric heterogeneity). Source label: Thomsen HIIT PsA RCT. |
| R06-E05 | R06 | Cardiorespiratory fitness | Cardiorespiratory fitness - VO2max | No-change/usual-care control | MD | 3.72 (2.38 to 5.06) | I² NR k 1 | Core-eligible · in primary | Analysis type: Narrative synthesis with extracted trial estimate. Native-scale estimate carried as reported; no cross-review pooling (overlap and metric heterogeneity). Source label: Thomsen HIIT PsA RCT. |
| R06-E06 | R06 | Cardiorespiratory fitness | Cardiorespiratory fitness - VO2max | No-change/usual-care control | MD | 3.08 (1.63 to 4.53) | I² NR k 1 | Core-eligible · in primary | Analysis type: Narrative synthesis with extracted trial estimate. Native-scale estimate carried as reported; no cross-review pooling (overlap and metric heterogeneity). Source label: Thomsen HIIT PsA RCT. |
| R06-E07 | R06 | Body composition | Body composition - truncal fat percentage | No-change/usual-care control | MD | -1.28 (-2.51 to -0.05) | I² NR k 1 | Core-eligible · in primary | Analysis type: Narrative synthesis with extracted trial estimate. Native-scale estimate carried as reported; no cross-review pooling (overlap and metric heterogeneity). Source label: Thomsen HIIT PsA RCT. |
| R06-E08 | R06 | Disease activity | Disease activity - BASDAI | Wait-list/usual-care control | Single-trial contrast (as reported) | -2/10 | I² NR k 1 | Core-eligible · in primary | Analysis type: Narrative synthesis with extracted trial result. Native-scale estimate carried as reported; no cross-review pooling (overlap and metric heterogeneity). Source label: Roger-Silva resistance-training RCT. |
| R06-E09 | R06 | Pain | Pain - VAS | Wait-list/usual-care control | Single-trial contrast (as reported) | -25.1/100 | I² NR k 1 | Core-eligible · in primary | Analysis type: Narrative synthesis with extracted trial result. Native-scale estimate carried as reported; no cross-review pooling (overlap and metric heterogeneity). Source label: Roger-Silva resistance-training RCT. |
| R06-E10 | R06 | Physical function | Physical function - HAQ | Wait-list/usual-care control | Single-trial contrast (as reported) | -0.27 | I² NR k 1 | Core-eligible · in primary | Analysis type: Narrative synthesis with extracted trial result. Native-scale estimate carried as reported; no cross-review pooling (overlap and metric heterogeneity). Source label: Roger-Silva resistance-training RCT. |
| R06-E11 | R06 | Quality of life | Pain-related quality of life - SF-36 bodily pain | Wait-list/usual-care control | Single-trial contrast (as reported) | 72.5 vs 53.4 at follow-up | I² NR k 1 | Core-eligible · in primary | Analysis type: Narrative synthesis with extracted trial result. Native-scale estimate carried as reported; no cross-review pooling (overlap and metric heterogeneity). Source label: Roger-Silva resistance-training RCT. |
| R06-E12 | R06 | Quality of life | Quality of life - SF-36 general health | Wait-list/usual-care control | Single-trial contrast (as reported) | 63.6 vs 53.0 at follow-up | I² NR k 1 | Core-eligible · in primary | Analysis type: Narrative synthesis with extracted trial result. Native-scale estimate carried as reported; no cross-review pooling (overlap and metric heterogeneity). Source label: Roger-Silva resistance-training RCT. |
| R06-E13 | R06 | Muscular fitness | Muscle strength - leg extension | Wait-list/usual-care control | Single-trial contrast (as reported) | NR | I² NR k 1 | Core-eligible · in primary | Analysis type: Narrative synthesis with extracted trial result. Native-scale estimate carried as reported; no cross-review pooling (overlap and metric heterogeneity). Source label: Roger-Silva resistance-training RCT. |
| R07-E01 | R07 | Fatigue | Fatigue - VAS | No-change/usual-care control | MD | -12.83 (-25.88 to 0.23) | I² NR k 1 | Evidence-map-only · map only | Partial PICOS match; contributes to evidence map only, not main conclusions. — Analysis type: Narrative synthesis (not included in meta-analysis). Native-scale estimate carried as reported; no cross-review pooling (overlap and metric heterogeneity). Source label: Narrative PsA physical activity/exercise result. |
| R07-E02 | R07 | Fatigue | Fatigue - VAS | No-change/usual-care control | MD | 5.20 (-8.00 to 18.41) | I² NR k 1 | Evidence-map-only · map only | Partial PICOS match; contributes to evidence map only, not main conclusions. — Analysis type: Narrative synthesis (not included in meta-analysis). Native-scale estimate carried as reported; no cross-review pooling (overlap and metric heterogeneity). Source label: Narrative PsA physical activity/exercise result. |
| R08-E01 | R08 | Disease activity | Disease activity - composite disease activity | No-change/usual-care control | Narrative | NR | I² NR k 1 | Evidence-map-only · map only | Partial PICOS match; contributes to evidence map only, not main conclusions. — Analysis type: Narrative synthesis. Native-scale estimate carried as reported; no cross-review pooling (overlap and metric heterogeneity). Source label: Thomsen HIIT PsA RCT. |
| R08-E02 | R08 | Pain | Pain - VAS | No-change/usual-care control | Narrative | NR | I² NR k 1 | Evidence-map-only · map only | Partial PICOS match; contributes to evidence map only, not main conclusions. — Analysis type: Narrative synthesis. Native-scale estimate carried as reported; no cross-review pooling (overlap and metric heterogeneity). Source label: Thomsen HIIT PsA RCT. |
| R08-E03 | R08 | Fatigue | Fatigue - VAS | No-change/usual-care control | MD | -12.83 (-25.88 to 0.23) | I² NR k 1 | Evidence-map-only · map only | Partial PICOS match; contributes to evidence map only, not main conclusions. — Analysis type: Narrative synthesis with extracted mean difference. Native-scale estimate carried as reported; no cross-review pooling (overlap and metric heterogeneity). Source label: Thomsen HIIT PsA RCT. |
| R08-E04 | R08 | Cardiorespiratory fitness | Cardiorespiratory fitness - VO2max | No-change/usual-care control | MD | 3.72 (2.38 to 5.06) | I² NR k 1 | Evidence-map-only · map only | Partial PICOS match; contributes to evidence map only, not main conclusions. — Analysis type: Narrative synthesis with extracted mean difference. Native-scale estimate carried as reported; no cross-review pooling (overlap and metric heterogeneity). Source label: Thomsen HIIT PsA RCT. |
| R08-E05 | R08 | Cardiorespiratory fitness | Cardiorespiratory fitness - VO2max | No-change/usual-care control | MD | 3.08 (1.63 to 4.53) | I² NR k 1 | Evidence-map-only · map only | Partial PICOS match; contributes to evidence map only, not main conclusions. — Analysis type: Narrative synthesis with extracted mean difference. Native-scale estimate carried as reported; no cross-review pooling (overlap and metric heterogeneity). Source label: Thomsen HIIT PsA RCT. |
| R08-E06 | R08 | Body composition | Body composition - truncal fat percentage | No-change/usual-care control | MD | -1.28 (-2.51 to -0.05) | I² NR k 1 | Evidence-map-only · map only | Partial PICOS match; contributes to evidence map only, not main conclusions. — Analysis type: Narrative synthesis with extracted mean difference. Native-scale estimate carried as reported; no cross-review pooling (overlap and metric heterogeneity). Source label: Thomsen HIIT PsA RCT. |
| R08-E07 | R08 | Body composition | Body composition - total body fat percentage | No-change/usual-care control | MD | -0.80 (-1.71 to 0.10) | I² NR k 1 | Evidence-map-only · map only | Partial PICOS match; contributes to evidence map only, not main conclusions. — Analysis type: Narrative synthesis with extracted mean difference. Native-scale estimate carried as reported; no cross-review pooling (overlap and metric heterogeneity). Source label: Thomsen HIIT PsA RCT. |
| R08-E08 | R08 | Body composition | Body mass index | No-change/usual-care control | MD | -0.31 (-0.78 to 0.17) | I² NR k 1 | Evidence-map-only · map only | Partial PICOS match; contributes to evidence map only, not main conclusions. — Analysis type: Narrative synthesis with extracted mean difference. Native-scale estimate carried as reported; no cross-review pooling (overlap and metric heterogeneity). Source label: Thomsen HIIT PsA RCT. |
| R08-E09 | R08 | Inflammation/imaging | Imaging - axial PsA inflammatory lesions | No-change/usual-care control | Narrative | NR | I² NR k 1-2 trial reports | Evidence-map-only · map only | Partial PICOS match; contributes to evidence map only, not main conclusions. — Analysis type: Narrative synthesis. Native-scale estimate carried as reported; no cross-review pooling (overlap and metric heterogeneity). Source label: Chronaiou/Thomsen PsA imaging and inflammation reports. |
| R08-E10 | R08 | Inflammation/imaging | Inflammation/imaging - peripheral and axial PsA | No-change/usual-care control | Narrative | NR | I² NR k 1-2 trial reports | Evidence-map-only · map only | Partial PICOS match; contributes to evidence map only, not main conclusions. — Analysis type: Narrative synthesis. Native-scale estimate carried as reported; no cross-review pooling (overlap and metric heterogeneity). Source label: Chronaiou/Thomsen PsA imaging and inflammation reports. |
| R09-E01 | R09 | Psoriasis prevalence associations | Physical activity level/intensity - overall | Healthy/control comparator | RR | 0.89 (0.78 to 1.03) | I² 82% k 13 observational studies (outcome-specific counts vary) | Core-eligible · in primary | Analysis type: Meta-analysis of observational studies. Native-scale estimate carried as reported; no cross-review pooling (overlap and metric heterogeneity). Source label: Psoriasis physical activity association. |
| R09-E02 | R09 | Psoriasis prevalence associations | Physical activity level/intensity - vigorous exercise | Healthy/control comparator | RR | 0.76 (0.67 to 0.85) | I² 0% k 13 observational studies (outcome-specific counts vary) | Core-eligible · in primary | Analysis type: Meta-analysis of observational studies. Native-scale estimate carried as reported; no cross-review pooling (overlap and metric heterogeneity). Source label: Psoriasis physical activity association. |
| R09-E03 | R09 | Psoriasis prevalence associations | Physical activity level - exercise duration | Healthy/control comparator | SMD | -0.15 (-0.35 to 0.06) | I² 73% k 13 observational studies (outcome-specific counts vary) | Core-eligible · in primary | Analysis type: Meta-analysis of observational studies. Native-scale estimate carried as reported; no cross-review pooling (overlap and metric heterogeneity). Source label: Psoriasis physical activity association. |
| R09-E04 | R09 | Psoriasis prevalence associations | Physical activity level - exercise score | Healthy/control comparator | SMD | 0.56 (-0.89 to 2.01) | I² 98% k 13 observational studies (outcome-specific counts vary) | Core-eligible · in primary | Analysis type: Meta-analysis of observational studies. Native-scale estimate carried as reported; no cross-review pooling (overlap and metric heterogeneity). Source label: Psoriasis physical activity association. |
| R09-E05 | R09 | Psoriasis prevalence associations | Physical activity level/intensity - within-psoriasis intensity comparison | Healthy/control comparator | RR | 0.40 (0.18 to 0.90) | I² 99% k 13 observational studies (outcome-specific counts vary) | Core-eligible · in primary | Analysis type: Meta-analysis of observational studies. Native-scale estimate carried as reported; no cross-review pooling (overlap and metric heterogeneity). Source label: Psoriasis physical activity association. |
| R09-E06 | R09 | Psoriasis prevalence associations | Physical activity level/intensity - control intensity comparison | Healthy/control comparator | RR | 0.90 (0.46 to 1.77) | I² 100% k 13 observational studies (outcome-specific counts vary) | Core-eligible · in primary | Analysis type: Meta-analysis of observational studies. Native-scale estimate carried as reported; no cross-review pooling (overlap and metric heterogeneity). Source label: Psoriasis physical activity association. |
| R09-E07 | R09 | Psoriasis prevalence associations | Physical activity level/intensity - control intensity comparison | Healthy/control comparator | RR | 1.09 (0.43 to 2.75) | I² 97% k 13 observational studies (outcome-specific counts vary) | Core-eligible · in primary | Analysis type: Meta-analysis of observational studies. Native-scale estimate carried as reported; no cross-review pooling (overlap and metric heterogeneity). Source label: Psoriasis physical activity association. |
| R09-E08 | R09 | Psoriasis prevalence associations | Physical activity level/intensity - control intensity comparison | Healthy/control comparator | RR | 1.04 (0.41 to 2.63) | I² 98% k 13 observational studies (outcome-specific counts vary) | Core-eligible · in primary | Analysis type: Meta-analysis of observational studies. Native-scale estimate carried as reported; no cross-review pooling (overlap and metric heterogeneity). Source label: Psoriasis physical activity association. |
| R09-E09 | R09 | Psoriasis prevalence associations | Physical activity level/intensity - control intensity comparison | Healthy/control comparator | RR | 0.64 (0.25 to 1.64) | I² 98% k 13 observational studies (outcome-specific counts vary) | Core-eligible · in primary | Analysis type: Meta-analysis of observational studies. Native-scale estimate carried as reported; no cross-review pooling (overlap and metric heterogeneity). Source label: Psoriasis physical activity association. |
| R09-E10 | R09 | Psoriasis prevalence associations | Physical activity level - regular exercise frequency | Healthy/control comparator | RR | 0.88 (0.82 to 0.95) | I² 0% k 13 observational studies (outcome-specific counts vary) | Core-eligible · in primary | Analysis type: Meta-analysis of observational studies. Native-scale estimate carried as reported; no cross-review pooling (overlap and metric heterogeneity). Source label: Psoriasis physical activity association. |

All extracted review-level estimates are retained here, including all rows removed from the streamlined main Table 2: direction-only or NR findings, duplicate reports of the same primary-study estimate, descriptive secondary contrasts, and evidence-map-only rows. Estimates are reproduced as reported by each review; no umbrella-level pooling was performed. Rows from the observational psoriasis-prevalence review include RR and standardised-mean-difference analyses. “In primary” identifies core-eligible-review estimates; “map only” identifies evidence-map-only rows. Effect IDs preserve traceability to the extraction dataset.

Abbreviations: CI, confidence interval; I², heterogeneity statistic; k, contributing primary studies; NR, not reported. Effect-metric abbreviations are as defined for main Table 2.

**Table S3. Risk-of-bias tools and rating rationale**

| **Review** | **Primary-study risk-of-bias tool(s) and AMSTAR 2 rating rationale** |
| --- | --- |
| R01 Björk et al. | Primary-study tool(s): Cochrane RoB 2.0. Prospective registration, broad database search, duplicate screening/extraction/RoB, an excluded-study list in the supplement, RoB 2.0, GRADE, heterogeneity assessment, and publication-bias assessment were reported. The main weakness was lack of extracted funding-source information for the included primary studies, so overall confidence was rated Moderate rather than High. |
| R02 Gwinnutt et al. | Primary-study tool(s): AMSTAR 2 for reviews; abbreviated Cochrane RoB tool for RCTs; QUIPS for observational studies. The review had a clear question, large structured searches, RoB tools, evidence tables, and GRADE-informed recommendations, but no prospective protocol/registration was identified and no excluded-study list with justifications was provided. The authors also stated that data extraction and RoB assessment were not performed in duplicate. Two AMSTAR-2 critical-domain failures led to Critically Low confidence. |
| R03 Hailey et al. | Primary-study tool(s): RoB 2 for RCTs; ROBINS-I planned but CASP cohort checklist used for NRS. The review had a registered PROSPERO protocol, a comprehensive multi-database search, duplicate selection and extraction, study characteristics, formal RoB assessment, and GRADE for disease activity. No citation-level excluded-study list with justifications was found; because this is an AMSTAR-2 critical domain, overall confidence was rated Low. |
| R04 Kaerts et al. | Primary-study tool(s): RoB 2; Newcastle-Ottawa Scale; modified RoB 2 domains for single-group interventions. The review used a prospective PROSPERO protocol, four databases, duplicate blinded screening, formal RoB tools, extensive characteristics tables, and a narrative synthesis appropriate to heterogeneity. No citation-level excluded-study list with reasons was found, which is a critical AMSTAR-2 weakness, so confidence was Low. |
| R05 Kaerts et al. | Primary-study tool(s): Newcastle-Ottawa Scale; Mendelian-randomization appraisal as reported. The review had a prospective PROSPERO protocol, a comprehensive four-database search, duplicate blinded screening, formal RoB appraisal, detailed study tables, and a narrative synthesis matched to outcome diversity. The absence of a citation-level excluded-study list with justifications was the main AMSTAR-2 critical flaw, leading to Low confidence. |
| R06 Kessler et al. | Primary-study tool(s): NR. The review defined a relevant question and used two independent reviewers, but it was not prospectively registered, used a very limited search strategy, did not report a formal RoB tool, and did not provide a citation-level excluded-study list. Multiple critical-domain failures led to Critically Low confidence. |
| R07 Santos et al. | Primary-study tool(s): Cochrane risk of bias tool. The review had a PROSPERO protocol, broad multi-database search, duplicate screening/extraction/RoB, study characteristics, Cochrane RoB, appropriate meta-analytic methods, heterogeneity assessment, and small-study/publication-bias checks when possible. No citation-level excluded-study list with justifications was found, so AMSTAR-2 confidence was Low. |
| R08 Sutherland et al. | Primary-study tool(s): Cochrane RoB 2.0; PEDro scale. The review was prospectively registered, searched five databases, used defined RCT eligibility criteria, extracted study characteristics, and applied RoB 2.0 plus PEDro. No citation-level excluded-study list with justifications was found; therefore, one critical AMSTAR-2 flaw led to Low confidence. |
| R09 Zheng et al. | Primary-study tool(s): Newcastle-Ottawa Scale (NOS). The review was registered and used NOS, but the search was limited to three databases and English-language reports, no citation-level excluded-study list was provided, RoB was not strongly integrated into interpretation, and publication bias/small-study effects were not formally tested despite being relevant to a meta-analysis. Multiple critical concerns led to Critically Low confidence. |

Primary-study risk-of-bias tools are those each review applied to its own included studies, transcribed from the source reports.

**Table S4. Certainty of the evidence by domain, with rationale**

| **Outcome domain** | **Certainty framework** | **Certainty (range)** | **Most conservative** | **Reviews** | **Rating provenance** |
| --- | --- | --- | --- | --- | --- |
| Quality of life | GRADE; descriptive (non-GRADE) | Very Low | Very Low | R01; R03; R04; R06 | Mixed: source-review-reported where available; otherwise overview-applied |
| Physical function | GRADE; descriptive (non-GRADE) | Low–Very Low | Very Low | R01; R02; R03; R04; R06 | Mixed: source-review-reported where available; otherwise overview-applied |
| Disease activity | GRADE | Low–Very Low | Very Low | R02; R03; R04; R06; R08 | Mixed: source-review-reported where available; otherwise overview-applied |
| Pain | GRADE | Low–Very Low | Very Low | R02; R03; R06; R08 | Overview-applied |
| Fatigue | GRADE | Low–Very Low | Very Low | R03; R04; R06; R07; R08 | Overview-applied |
| Cardiorespiratory fitness | GRADE; descriptive (non-GRADE) | Moderate–Very Low | Very Low | R03; R04; R05; R06; R08 | Overview-applied |
| Body composition | GRADE | Low–Very Low | Very Low | R03; R04; R06; R08 | Overview-applied |
| PsA incidence / structural associations | Descriptive (non-GRADE) | Low–Very Low | Very Low | R05 | Overview-applied |
| Muscular fitness | GRADE; descriptive (non-GRADE) | Low–Very Low | Very Low | R05; R06 | Overview-applied |
| Physical activity level/behaviour | Descriptive (non-GRADE) | Low–Very Low | Very Low | R05; R06 | Overview-applied |
| Inflammation/imaging | GRADE | Very Low | Very Low | R08 | Overview-applied |
| Psoriasis prevalence associations | Ioannidis class (observational) | Class III; Class IV; No class (P≥0.05) | No class (P≥0.05) | R09 | Overview-applied |

Rating provenance is shown explicitly. Of 87 effect-level ratings, 81 were applied by the overview team and six were retained from source reviews (three from Björk et al. and three from Hailey et al.). Source-review GRADE ratings were retained only when they addressed the same construct, comparator, and time point. Otherwise, two overview reviewers independently applied GRADE de novo using review-reported information on risk of bias, inconsistency, indirectness, imprecision, and publication bias; duplicate review reports inherited the same construct-level judgement. Narrative-only findings received labelled descriptive, non-GRADE judgements. The overview reviewers applied Ioannidis class I–IV criteria to observational psoriasis-prevalence associations; unavailable bias inputs prevented higher classes. Disagreements were resolved by discussion and, if necessary, third-reviewer adjudication.

**Table S5. Corrected-covered-area verification**

| **Scope** | **Exercise/activity cluster or scenario** | **Inclusions** | **Unique studies** | **Reviews** | **CCA** | **Check** | **Interpretation** |
| --- | --- | --- | --- | --- | --- | --- | --- |
| Overall | All included reviews | 378 | 311 | 9 | 0.026929 | Matched | Slight overlap. |
| Cluster | Home-based exercise (R02 only) | 4 | 4 | 1 | Not computable | N/A | Single-review cluster. |
| Cluster | Lifestyle / passive physical-therapy boundary | 14 | 13 | 4 | 0.025641 | Matched | Slight overlap. |
| Cluster | Other exercise or rehabilitation | 59 | 53 | 4 | 0.037736 | Matched | Slight overlap. |
| Cluster | HIIT / cardiorespiratory training | 12 | 6 | 7 | 0.166667 | Matched | Very high overlap; Thomsen/Chronaiou trial family represented in seven reviews. |
| Cluster | Resistance / strength training | 149 | 111 | 7 | 0.057057 | Matched | Moderate overlap; Roger-Silva trial represented in six reviews. |
| Cluster | Fatigue / non-pharmacological physical-activity behaviour | 84 | 74 | 4 | 0.045045 | Matched | Slight overlap. |
| Cluster | Physical-activity level / fitness descriptive evidence | 56 | 50 | 4 | 0.040000 | Matched | Slight overlap. |
| Sensitivity scenario | Two potentially related Sveaas reports treated as one study | 377 | 310 | 9 | 0.027419 | Not primary | Overall classification remains slight; no conclusion changes. |

The corrected covered area (CCA) was computed as (N − r) / (r×c − r), where N is the number of inclusions, r the number of unique studies, and c the number of reviews. Each value was independently recomputed from the study-by-review citation matrix. The HIIT/cardiorespiratory cluster was driven by four linked reports from the Thomsen/Chronaiou parent trial (NCT02995460), and the resistance cluster was partly driven by the Roger-Silva 2018 trial (NCT02598739). The Silva 2023 active-comparator trial (NCT04304326) was retained as independent. The two potentially related Sveaas reports were retained as separate publications in the primary matrix; treating them as one study changed the overall CCA only from 0.026929 to 0.027419, with the classification remaining slight and no conclusion changing.

**Table S6. Exercise programmes underpinning the PsA intervention evidence**

| **Primary study / trial family** | **Population and design** | **Exercise programme** | **Comparator** | **Outcomes and overlap note** | **Trial registration / identity** |
| --- | --- | --- | --- | --- | --- |
| Thomsen/Chronaiou HIIT trial family (2018–2023) | PsA; 67 randomised in the parent trial; 61 contributed to the body-composition report and 39 to the axial-MRI subset. | 11 weeks; 3 sessions/week. Ten-minute warm-up, four 4-minute intervals at 85–95% HRmax, 3-minute active recovery at about 70%; two supervised stationary-cycle sessions and one self-directed session/week. | No-change/usual-care control | VO₂max, body composition, fatigue, disease activity, and imaging. Four linked reports were represented across seven reviews and counted as one parent trial when interpreting effects; harms reporting was incomplete. | NCT02995460. Thomsen 2018, Thomsen 2019, Chronaiou 2022, and Thomsen 2023 are linked reports from the same Norwegian parent trial; sample-size differences reflect outcome-specific subsets. |
| Roger-Silva et al. (2018) | PsA; randomised controlled trial; n=41. | Progressive resistance exercise twice weekly for 12 weeks. | Conventional drug treatment / wait-list control | BASDAI, HAQ, BASFI, pain, SF-36, and strength. The trial was represented in six reviews; no adverse events were reported in the PsA-specific lifestyle review. | NCT02598739. Independent from the later Silva 2023 active-comparator trial. |
| Silva et al. (2023) | PsA; active-comparator randomised trial; n=41. | Elastic-band functional exercise versus machine-based resistance exercise, twice weekly for 12 weeks; three sets of 12 repetitions. | Alternative active exercise programme | BASFI, HAQ-S, BASDAI, DAS28, SF-36, and strength. This trial appeared in two reviews. | NCT04304326. Distinct later trial with a different registration, comparator, and recruitment period; not a second report of Roger-Silva 2018. |
| Chimenti et al. (2014) | PsA; single-arm home-exercise study. | Twelve-week home programme; exercise dose was incompletely reported in the eligible reviews. | No concurrent comparator | Pain, function, and quality of life. Interpretation is limited by design and incomplete programme reporting. | Registration not reported in the included reviews. |

Programme details and trial identities were checked against the primary reports and registrations used to distinguish repeated reports from independent trials. They are provided to make exercise type, intensity, frequency, duration, and overlap clinically interpretable and do not constitute de novo effect synthesis.

**Table S7. Review-by-review evidence-tier classification**

Core-eligible status required all three primary-scope criteria: a psoriasis- or PsA-specific review question and synthesis, exercise/physical activity/fitness as the primary index construct, and at least one extractable prespecified disease-specific outcome. Reviews with relevant subgroup data but a mixed-disease or broader-intervention primary scope were retained for evidence mapping only.

| **Review** | **Disease-specific primary scope** | **Exercise / PA / fitness as primary index** | **Extractable prespecified outcome** | **Tier and determining reason** |
| --- | --- | --- | --- | --- |
| R01 Björk et al. | No — mixed RA/SpA/PsA review; PsA was a small subgroup. | Yes | Yes, but limited to a small PsA subgroup. | Evidence-map-only: mixed-disease primary scope. |
| R02 Gwinnutt et al. | No — seven RMDs. | Partial — exercise and body weight. | Yes for selected PsA rows. | Evidence-map-only: mixed-disease and broader-exposure scope. |
| R03 Hailey et al. | Yes — PsA. | No — broad lifestyle and non-pharmacological interventions. | Yes | Evidence-map-only: broader-intervention primary scope. |
| R04 Kaerts et al. (2024) | Yes — PsA. | Yes — physical therapy with active exercise programmes. | Yes | Core-eligible. |
| R05 Kaerts et al. (2025) | Yes — PsA. | Yes — physical fitness and physical activity. | Yes | Core-eligible. |
| R06 Kessler et al. | Yes — PsA. | Yes — physical activity and structured exercise. | Yes | Core-eligible. |
| R07 Santos et al. | No — mixed inflammatory RMDs. | No — broad non-pharmacological fatigue management. | PsA-specific quantitative synthesis was not separately available. | Evidence-map-only: mixed-disease and broader-intervention scope. |
| R08 Sutherland et al. | No — RA, PsA, and axSpA. | Yes — HIIT. | Yes for linked PsA reports. | Evidence-map-only: mixed-disease primary scope. |
| R09 Zheng et al. | Yes — cutaneous psoriasis. | Yes — physical-activity exposure. | Yes — psoriasis prevalence associations. | Core-eligible. |

**Table S8. Safety and adverse-event reporting across included reviews**

Adverse events, flares, and safety outcomes were listed as additional outcomes in the PROSPERO record. Reporting across the included reviews was sparse, inconsistent, and often not PsA-specific; therefore, findings are summarised narratively and no comparative risk estimate or disease-specific safety conclusion is made.

| **Review** | **Safety-related reporting** | **PsA-specific details** | **Completeness / interpretation** | **Use in this umbrella review** |
| --- | --- | --- | --- | --- |
| R01 Björk et al. | No dedicated PsA adverse-event synthesis. | No comparative PsA harms estimate. | Insufficient for a disease-specific conclusion. | Context only. |
| R02 Gwinnutt et al. | No dedicated PsA comparative safety synthesis. | PsA-specific adverse-event denominators were not reported. | Insufficient for a disease-specific conclusion. | Context only. |
| R03 Hailey et al. | Six of eight included studies did not report adverse events. | Roger-Silva 2018 reported no adverse events. The HIIT report described one withdrawal after a stroke and difficulty tolerating the programme, with no other adverse events reported. | Substantial under-reporting; no comparative risk estimate. | Narrative safety summary. |
| R04 Kaerts et al. (2024) | HIIT, resistance, and functional training were described as well tolerated and without deleterious disease-activity changes. | No complete adverse-event denominator or comparative harm estimate was provided. | Stable disease activity is not equivalent to comprehensive safety assessment. | Context only. |
| R05 Kaerts et al. (2025) | Not primarily an intervention-safety review. | No systematic PsA harms synthesis. | Not informative for intervention safety. | Not used for a safety conclusion. |
| R06 Kessler et al. | Two more recent PsA trials reported no exercise-related adverse events or inflammatory flares. | An older mixed PsA/recent-onset RA trial reported temporary inflammatory flares in 6 exercise versus 5 control participants and acute low-back pain in 1 participant per group. | Mixed populations and incomplete reporting preclude a PsA-specific estimate. | Narrative safety summary. |
| R07 Santos et al. | Safety data were reported in 31 mixed-I-RMD RCTs, but most studies did not address safety. | PsA-specific data were not separable. | Reassuring mixed-disease statements cannot establish PsA safety. | Context only. |
| R08 Sutherland et al. | HIIT was described as safe mainly because disease activity did not worsen. | No complete PsA adverse-event synthesis or denominator was provided. | No disease-specific comparative risk estimate. | Context only. |
| R09 Zheng et al. | Observational prevalence meta-analysis. | Not applicable. | Does not assess intervention harms. | Not applicable. |
